# Schwann cells are axo-protective after injury irrespective of myelination status in mouse Schwann cell–neuron cocultures

**DOI:** 10.1242/jcs.261557

**Published:** 2023-09-20

**Authors:** Clara Mutschler, Shaline V. Fazal, Nathalie Schumacher, Andrea Loreto, Michael P. Coleman, Peter Arthur-Farraj

**Affiliations:** ^1^ John Van Geest Centre for Brain Repair, Department of Clinical Neurosciences, University of Cambridge, Cambridge CB2 0PY, UK; ^2^ Wellcome-MRC Cambridge Stem Cell Institute, University of Cambridge, Cambridge CB2 0AW, UK; ^3^ Laboratory of Nervous System Disorders and Therapies, GIGA Neurosciences, University of Liège, 4000 Liège, Belgium

**Keywords:** Mouse, Schwann cell, Dorsal root ganglion neuron, Myelination, Coculture, Axon degeneration, Wallerian degeneration

## Abstract

Myelinating Schwann cell (SC)–dorsal root ganglion (DRG) neuron cocultures are an important technique for understanding cell–cell signalling and interactions during peripheral nervous system (PNS) myelination, injury, and regeneration. Although methods using rat SCs and neurons or mouse DRG explants are commonplace, there are no established protocols for compartmentalised myelinating cocultures with dissociated mouse cells. There consequently is a need for a coculture protocol that allows separate genetic manipulation of mouse SCs or neurons, or use of cells from different transgenic animals to complement *in vivo* mouse experiments. However, inducing myelination of dissociated mouse SCs in culture is challenging. Here, we describe a new method to coculture dissociated mouse SCs and DRG neurons in microfluidic chambers and induce robust myelination. Cocultures can be axotomised to study injury and used for drug treatments, and cells can be lentivirally transduced for live imaging. We used this model to investigate axon degeneration after traumatic axotomy and find that SCs, irrespective of myelination status, are axo-protective. At later timepoints after injury, live imaging of cocultures shows that SCs break up, ingest and clear axonal debris.

## Teaser

**Highlighted Article:** A novel method for compartmentalised mouse myelinating Schwann cell–DRG neuron cocultures, showing that Schwann cell myelination status does not influence their axon protective effect after injury.

## INTRODUCTION

Dissociated myelinating Schwann cell (SC)–dorsal root ganglion (DRG) cocultures from rats were first developed by the Bunge laboratory in the 1980s to investigate peripheral nervous system (PNS) myelination in a more dynamic way (Bunge et al., 1989; Eldridge et al., 1987). These cultures have been used to make seminal discoveries in uncovering the cellular and molecular mechanisms of SC myelination alongside *in vivo* investigation. These include how the inner SC membrane (mesaxon) advances to myelinate axons, and the role of β-neuregulin-1 (βNRG1) and polarity proteins in SC myelination (Bunge et al., 1989; Chan et al., 2006; Shen et al., 2014; Taveggia et al., 2005). Similarly, SC–DRG cocultures have been useful in demonstrating how SCs proliferate after axon injury, transfer metabolites, such as pyruvate, to delay axon degeneration, how placental growth factor (Plgf) regulates axon fragmentation by SCs and how SC JUN promotes axon outgrowth after injury (Arthur-Farraj et al., 2011; Babetto et al., 2020; Salzer and Bunge, 1980; Vaquié et al., 2019). The use of a coculture system to study axon–SC interactions during axon degeneration and regeneration offers some advantages over *in vivo* approaches as both neurons and SCs can be genetically manipulated separately and live imaged with ease. Although there are methodological descriptions available for preparing dissociated cocultures using rat SCs with either rat or mouse DRG neurons, in addition to fully compartmentalised rat cocultures, and mouse explant cocultures, there are no current published protocols for establishing fully dissociated and compartmentalised mouse myelinating cocultures (Taveggia and Bolino, 2018; Vaquié et al., 2018). Indeed there has only ever been one laboratory detailing convincing myelin formation in dissociated mouse myelinating SC–DRG neuron cocultures; however, this was never published as a step-by-step detailed protocol (Stevens and Fields, 2000; Stevens et al., 1998). In the past 20 years, there have been no published studies demonstrating myelination in fully dissociated mouse SC–mouse DRG cocultures. This has largely prevented the use of cells, particularly SCs, from transgenic mice in cocultures and thus restricted the ability to study SC–axon interactions in a system that can be readily manipulated and live imaged with results directly applied back to *in vivo* findings in the same species.

The consensus within the field is that inducing myelination in dissociated mouse SCs is challenging. Certainly, induction of myelin differentiation with cyclic adenosine monophosphate (cAMP) analogues or elevating agents, such as forskolin, is more difficult in mouse SC monocultures compared to rat SC cultures. This is because mouse SCs require additional exogenous βNRG1, plating on poly-L-lysine (PLL) instead of poly-D-lysine (PDL), and low concentration horse serum (HS) as opposed to fetal calf serum (Arthur-Farraj et al., 2011; Päiväläinen et al., 2008; Stevens et al., 1998). Protocols exist where endogenous mouse SCs are used to myelinate dissociated or non-dissociated DRG explant cultures. (Harty et al., 2019; Numata-Uematasu et al., 2023; Shen et al., 2014; Stettner et al., 2013; Sundaram et al., 2021). Furthermore, another protocol uses exogenous SCs seeded onto non-dissociated DRG explant cultures (Päiväläinen et al., 2008). Other laboratories seed cultured rat SCs onto dissociated mouse DRG axons (Taveggia and Bolino, 2018). Use of dissociated or non-dissociated DRG explant cultures precludes many experimental uses, such as using SCs from different transgenic animals and separate transduction of SCs and neurons with viruses for live imaging or genetic manipulation. Additionally, explant cultures impede use of microfluidic chambers to allow injury studies and separate drug treatments to neurons or SCs. The reason for this is that antimitotics cannot be used in dissociated or non-dissociated DRG explant cultures as this depletes SCs, and the culture consequently quickly becomes contaminated with other non-neuronal cell types, such as satellite cells and fibroblasts migrating out of the DRG. Furthermore, use of exogenous SCs in a non-dissociated DRG explant culture, after a period of antimitotic exposure, as developed by Päiväläinen et al. (2008), still risks potential contamination from endogenous SCs and satellite glia migrating out of the DRG explant over time. This occurs because antimitotic treatment is unlikely to fully penetrate the whole DRG without prior dissociation. Additionally, a compartmentalised culture system cannot be readily used with non-dissociated DRG explant cultures (Päiväläinen et al., 2008).

Recent studies using SC–DRG cocultures to investigate axon–SC interactions after injury have found differing results. A study using dissociated rat myelinating SC–DRG neuron cocultures in microfluidic chambers found that the presence of SCs accelerated the disintegration of axons after traumatic axotomy at late timepoints (Vaquié et al., 2019). A second study seeded rat SCs on mouse DRG axons, in microfluidic chambers in short-term culture, but did not induce them to myelinate, and they found that the presence of SCs delayed axon degeneration (Babetto et al., 2020). Certainly data from *in vivo* studies, first in zebrafish and later in mouse, have shown that SCs do participate in the breakup of the axon (Catenaccio et al., 2017; Rosenberg et al., 2014; Vaquié et al., 2019; Villegas et al., 2012). It is possible that the different conclusions of the two coculture studies could be confounded by a species difference, given that Babetto et al. (2020) combined rat and mouse cells whereas Vaquié et al. (2019) studied solely rat cells. Another explanation is that the myelination status of the SCs may influence the outcome of the experiment as Vaquié et al. (2019) induced myelination prior to injury whereas Babetto et al. (2020) did not. Given these outstanding questions, we investigated whether SCs accelerate or delay axon degeneration, and whether the outcome depends upon myelination status, in a fully mouse SC–DRG coculture system.

We describe a detailed protocol for setting up dissociated mouse myelinating SC–DRG neuron cocultures in microfluidic chambers. We demonstrate how these compartmentalised cocultures can be used for performing axotomies, drug treatments, lentiviral infection of DRG neurons and SCs separately, and live imaging. SCs can be induced to robustly express the myelin markers periaxin (PRX), myelin protein zero (MPZ) and myelin basic protein (MBP), and form electron-dense myelin and nodal and paranodal structures. Additionally, SCs that align with axons and are not induced to myelinate appear to ensheath multiple axons. After axotomy, cocultured SCs replicate key parts of the *in vivo* injury response, upregulating the major injury transcription factor, JUN (also known as c-JUN), demyelinating and forming myelin ovoids (Arthur-Farraj and Coleman, 2021; Arthur-Farraj et al., 2012). We then show that after traumatic axotomy, SCs first have an axo-protective role, as severed DRG axons in the presence of SCs degenerate more slowly, compared to severed DRG axons cultured on their own. We find that both myelinating SCs and aligned SCs, which have not been induced to myelinate, are capable of delaying axon degeneration, suggesting that myelination status is not important in regulating this phenomenon. At later timepoints after axotomy, live imaging of cultures reveals that SCs help break up and ingest axonal fragments, clearing the debris.

## RESULTS

### Establishing dissociated mouse myelinating SC–DRG neuron cocultures in microfluidic chambers

**Fig. 1.**
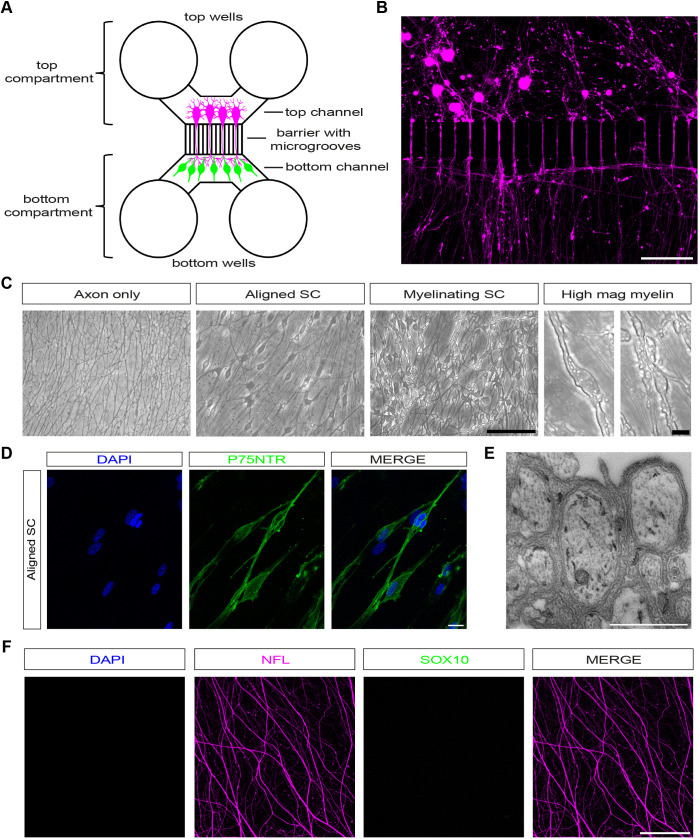
**Dissociated murine SC–DRG neuron cocultures in microfluidic chambers.** (A) Standard neuron device with a 150 μm microgroove barrier. Dissociated DRG neurons (magenta), which cannot cross the barrier owing to their size, are seeded into the top channel and extend axons into the bottom channel, reaching the bottom wells. SCs (green) are then seeded in the bottom channel where they align with and myelinate axons. (B) Dissociated DRG neurons (magenta) growing across the microgroove barrier. Scale bar: 100 μm. (C) Phase images of the bottom channel showing axons only, axons with aligned SCs (aligned SC), and axons with myelinating SCs (myelinating SC). Scale bar: 100 μm. Higher magnification images show myelin segments in cultures with myelinating SCs. Scale bar: 10 μm. (D) Aligned SCs can be labelled with p75NTR. Scale bar: 20 μm. (E) Electron micrographs of aligned Schwann cells that show structures resembling Remak bundles. Scale bar: 1 nm. (F) Confocal images of axon-only cultures, showing axons (NFL, magenta), but no DAPI (blue) or SOX10 (green) signal in the axonal compartment of the microfluidic chamber. Scale bar: 100 μm. All images representative of at least three experimental repeats.

To establish dissociated mouse myelinating SC–DRG neuron cocultures, we dissected DRGs from embryonic day (E)14 mice, enzyme dissociated these (see Materials and Methods), and seeded the DRG neuronal cell suspension into the top compartment of microfluidic chambers on PLL- and Matrigel^®^-coated Aclar^®^ coverslips (Fig. 1A). DRG neurons are then purified using the anti-mitotic cytosine Arabinoside (Ara-C) and are allowed to extend axons across a 150 μm microgroove barrier into an axonal compartment for up to 7 days (Fig. 1B). Owing to their size, DRG neurons cannot cross this microgroove barrier, and are exclusively present in the top compartment of the microfluidic chamber. By maintaining a hydrostatic pressure gradient between the two compartments, we were able to apply this anti-mitotic solely to the DRG compartment. Postnatal day 2–4 (P2–P4) neonatal sciatic nerves were then dissected, enzyme dissociated and Ara-C purified in DMEM with 5% HS for 72 h to obtain cultured mouse SCs (Arthur-Farraj et al., 2011). Ara-C was withdrawn from microfluidic chambers for 48 h before cultured mouse SCs were seeded into the axonal compartment of the chambers, where they were allowed to align with axons and proliferate for up to 1 week (Fig. 1C). SCs could then be induced to myelinate over the course of approximately 3 weeks through supplementation of their cell culture medium with forskolin (10 μM), βNRG1 (10 ng ml^−1^), Matrigel^®^ (1:100) and L-ascorbic acid (50 μg ml^−1^; Fig. 1C). Importantly, we found that L-ascorbic acid was insufficient to induce substantial myelination in our cultures, unlike in rat SC–DRG cocultures, and in the one previously published dissociated mouse SC–DRG protocol (Stevens et al., 1998). In fact, plating cocultures on laminin, adding ascorbic acid (50 μg ml^−1^), βNRG1 (10 ng ml^−1^) and forskolin (10 μM) induced very few myelin sheaths (Fig. S1). Only when cultures were plated on Matrigel^®^ and further Matrigel^®^ was added to the myelination medium for each medium change, were we able to visualise robust reproducible myelination in our cocultures (Fig. S1). Forskolin and βNRG1 were included in the myelination medium, as we have previously shown that these agents can induce myelin proteins in cultured mouse SCs (Arthur-Farraj et al., 2011). For a detailed step-by-step description of how to set up these cultures please see the Materials and Methods section.

**Fig. 2.**
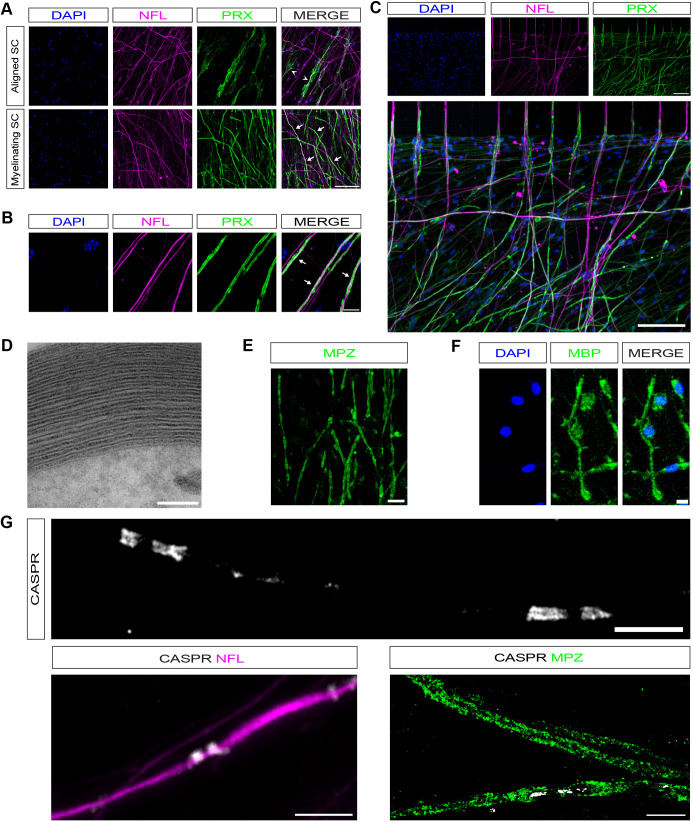
**SCs in dissociated myelinating cocultures can be induced to robustly myelinate.** (A) Confocal images of dissociated DRG neuron cocultures with aligned SC or myelinating SC. Axons are NFL-labelled (magenta), and SCs PRX-labelled (green). Aligned SCs have a more diffuse morphology (white arrowheads), while myelin segments are present in myelinating SCs (white arrows). Scale bar: 100 μm. (B) Higher magnification images showing NFL-labelled axons (magenta) covered by myelin segments (PRX, green, white arrows). Scale bar: 10 μm. (C) DRG neurons (NFL, magenta) growing across the barrier in chambers with SCs (PRX, green) that have been induced to myelinate. Scale bar: 100 μm. (D) Electron micrograph of electron-dense myelin in cocultures with myelinating SCs. Scale bar: 100 nm. (E) MPZ (green)-labelled myelinating SCs. Scale bar: 20 μm. (F) MBP (green)-labelled myelinating SCs. Scale bar: 10 μm. (G) In mature myelinating cultures, SCs CASPR (white) can be detected in the characteristic staining pattern marking paranodes. Scale bar 5 μm. Paranodal CASPR co-localised on axons (magenta). Scale bar: 5 μm. When colabelling with MPZ (green), CASPR is localised to paranodal loops adjacent to a node of Ranvier. Scale bar: 10 μm. All images representative of at least three experimental repeats.

After ∼6 weeks in culture, we were able to generate cultures with only axons in the axonal compartment, cultures with SCs aligned to axons but not induced to myelinate (aligned SC), and cultures with SCs myelinating axons (myelinating SC; Fig. 1C). Myelinating cocultures and cultures with aligned SCs can be easily distinguished by phase contrast microscopy, as myelin segments could be identified as long-phase bright structures surrounding axons (Fig. 1C). In cultures with aligned SCs, they labelled readily with p75NTR (also known as NGFR), and electron microscopy (EM) analysis demonstrated SCs ensheathing multiple axons (Fig. 1D,E). A total of 7 days of pulsed Ara-C treatment to the top compartment removed any non-neuronal cells. To confirm that axon-only cultures are not contaminated by any potential surviving endogenous SCs that have migrated from the top compartment, we show that there was no DAPI- or SC-specific SOX10 nuclear staining in the axonal compartment (Fig. 1F). In cocultures with aligned and myelinating SCs, we labelled SCs with antibodies against the myelin-associated protein periaxin (PRX), which is expressed in the mouse sciatic nerve around birth, at the initiation of myelination (Gillespie et al., 1994). In cultures with aligned SCs, these cells have a much more diffuse spread-out morphology, and a subset of these cells express PRX, whereas in myelinating cultures, strongly PRX-positive myelin segments are present (Fig. 2A–C). When quantifying the number of PRX-positive myelin segments, we found that there were 325.33±12.3 (mean±s.e.m.) sheaths per mm^2^, which is comparable to what has been originally described in rat SC–DRG cocultures and two-fold more extensive myelination than in recently described compartmentalised rat cocultures models (Eldridge et al., 1987; Vaquié et al., 2019). Furthermore, 25.47±1% (mean±s.e.m.) of SCs were myelinating in our cultures (*n*=3; Table 1). To confirm that cocultured myelinated SCs formed compact myelin, we performed EM, which revealed compact myelin formation with multiple myelin wraps and formation of readily visible major dense (MDL) and intraperiod lines (IPL; Fig. 2D). Additionally, we measured the periodicity, i.e. the distance between two adjacent major dense lines, to make sure myelin was compacted. Interperiodic distance was 12.16±0.28 nm (mean±s.e.m.), in line with previous reports (*n*=3; Table 1; Boutary et al., 2021; Fernando et al., 2016; García-Mateo et al., 2018; Giese et al., 1992; Perrot et al., 2007). Additionally, we immunolabelled cultures with antibodies against compact myelin proteins and found myelin sheaths were positive for MPZ and MBP (Fig. 2E,F). As myelinated fibres are organised into distinct domains, including the node of Ranvier, paranodal and juxtaparanodal regions, and the internode, we wanted to confirm whether contactin-associated protein 1 (CASPR1, also known as CNTNAP1, neurexin IV or paranodin) is confined to paranodal regions, where it normally accumulates in mature sheaths (Einheber et al., 1997; Salzer, 2015). We detected CASPR1 protein in the correct distribution, labelling the paranodal region, adjacent to regions of MPZ labelling (indicating compact myelin) in our myelinating SC–DRG neurons cocultures (Fig. 2G).

**Table 1.**
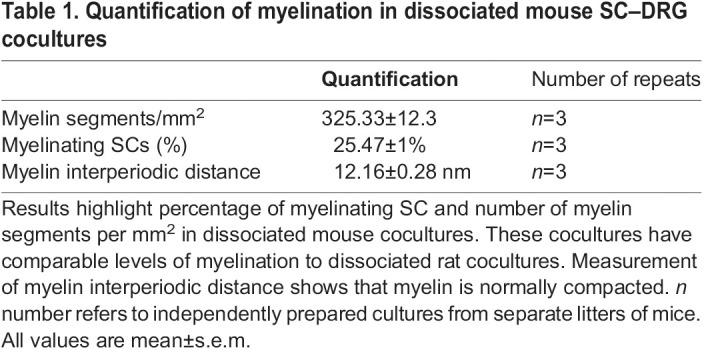
Quantification of myelination in dissociated mouse SC–DRG cocultures

In summary, our dissociated mouse myelinating SC–DRG neuron cocultures develop robust compact myelin, as evidenced by myelin protein immunostaining and EM. Our cocultures also develop nodal and paranodal structures, and the myelination efficiency is comparable to dissociated rat SC–DRG neurons cocultures.

### Axotomy in SC–DRG neuron cocultures replicates characteristic axonal and SC injury responses

**Fig. 3.**
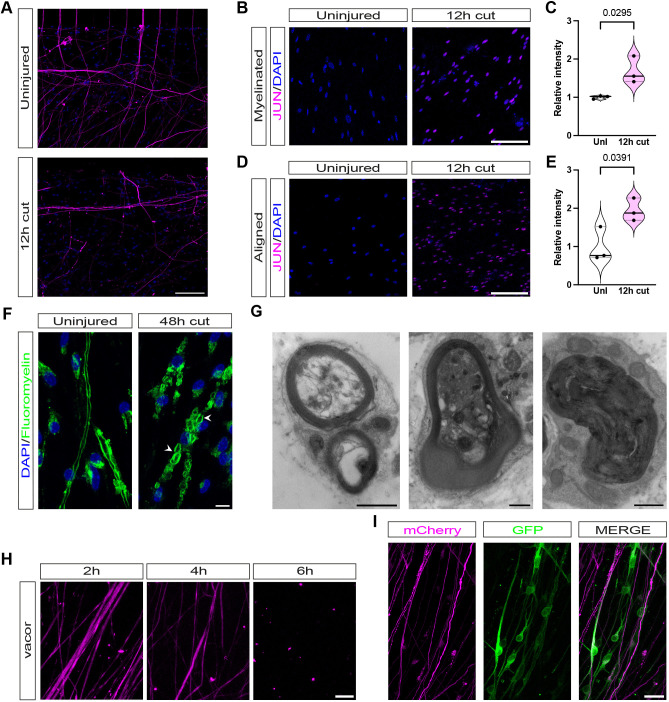
**SC–DRG neuron cocultures replicate characteristic axonal and SC injury responses after axotomy.** (A) At 12 h post axotomy many of the axons (magenta) in SC–DRG neuron cocultures have degenerated. Scale bar: 100 μm. (B) SC JUN in uninjured cultures and 12 h post axotomy. With identical imaging conditions, a signal can only be detected 12 h post axotomy. Scale bar: 100 μm. (C) Relative intensity of JUN signal in uninjured myelinating cultures and 12 h post axotomy (*n*=3). Data shown in violin plots with median (line) and upper and lower quartiles (dotted lines) marked. (D) SC JUN in uninjured cultures with aligned Schwann cells and 12 h post axotomy. With identical imaging conditions, a signal can only be detected 12 h post axotomy. Scale bar 100 μm. (E) Relative intensity of JUN signal in uninjured cultures with aligned Schwann cells and 12 h post axotomy (*n*=3). Data shown in violin plots with median (line) and upper and lower quartiles (dotted lines) marked. Statistical analysis in C and E was performed with two-tailed unpaired Student's *t*-test. *P* values are shown in the figure. (F) Myelinating SCs demyelinate after extended periods of time (48 h) after axotomy. Myelin ovoids and myelin debris (both identified by white arrowheads) are present in fluoromyelin (green)-labelled cultures. Scale bar: 10 μm. (G) Electron micrographs of myelinating SCs at 48 h post axotomy showing characteristic demyelinated profiles surrounding degenerated axons. Scale bars: 1 μm. (H) Vacor induces neurodegeneration when applied to the top compartment of cocultures. NFL (magenta). Scale bar: 10 μm. (I) Neurons and SCs can be lentivirally infected prior to plating in microfluidic chambers. Neurons were infected with LV-CMV-mCherry (MOI 2, magenta) and SCs with LV-CMV-GFP (MOI 200, green). Scale bar: 20 μm. *n* number refers to independently prepared cultures from separate litters of mice. All images representative of at least three experimental repeats.

After injury, SCs transform into repair SCs, which are characterised by a strong upregulation of the transcription factor JUN, and myelin breakdown by SCs through a process termed myelinophagy (Arthur-Farraj et al., 2012; Gomez-Sanchez et al., 2015; Jessen and Arthur-Farraj, 2019; Parkinson et al., 2008). We wanted to test whether axons and SCs respond to injury in a similar way in our coculture model as they do *in vivo*. Thus, we performed axotomies on cultures, using a scalpel under a light microscope to cut axons at the level of the microfluidic barrier, after carefully removing the chamber. In myelinating SC–DRG cocultures, 12 h after axotomy, we fixed and immunolabelled cultures for neurofilament light chain (NFL, also known as NEFL) and found that many axons distal to the site of axotomy had started to degenerate (*n*=4; Fig. 3A). Additionally, we noted a strong upregulation of JUN protein in SCs 12 h after axotomy (Fig. 3B,C). We also saw significant JUN upregulation 12 h after axotomy in cocultures with aligned SCs (Fig. 3D,E). Fluoromyelin labelling at 48 h post axotomy demonstrated myelin-ovoid formation, suggestive of active SC demyelination (Jung et al., 2011; Fig. 3F). We confirmed SC demyelination in our axotomised cocultures using EM, identifying characteristic demyelinated profiles surrounding degenerated axons, similar to what has been previously shown in rat SC–DRG cocultures (Fernandez-Valle et al., 1995; Fig. 3G).

In addition to traumatic axotomy, our cocultures can also be used for drug treatments. To demonstrate this, we treated only DRG cell bodies with the specific SARM1 agonist vacor, which induces specific degeneration of axons and neuronal cell bodies but not of SCs, which are completely insensitive to SARM1 agonists (Fazal et al., 2023; Loreto et al., 2021). 50 μM vacor addition to DRG cell bodies induced axon degeneration within 6–8 h in our cocultures (*n*=4, Fig. 3H). Finally, we also show that DRG neurons and SCs can be separately infected with lentiviruses (LVs) to permit live-cell imaging. Here, we infected mouse DRGs directly after the DRG dissociation step with an mCherry-expressing LV (LV-CMV-mCherry) and mouse SCs were infected with a GFP-expressing LV (LV-CMV-GFP) after Ara-C purification and prior to seeding in the microfluidic chamber (Fig. 3I). We found both mouse DRGs and SCs transduced best with LVs in suspension with slow centrifugation (see Materials and Methods). Importantly dissociated mouse SCs required a much higher multiplicity of infection (MOI) than dissociated mouse DRGs (see Materials and Methods). Interestingly, we found that embryonic DRG neurons were almost completely resistant to LV transduction if they had already been cultured for 2–3 days (data not shown).

In summary, our mouse myelinating SC–DRG cocultures can be axotomised to study SC–axon interactions, as they reliably replicate *in vivo* cellular behaviours. Furthermore, these cocultures can be used to study drug-induced neurodegeneration and both DRG neurons and SCs can be separately transduced with LVs for live imaging and genetic disruption studies.

### At early timepoints after axotomy, SCs are axo-protective independently of myelination status, and at later timepoints they clear axonal debris

**Fig. 4.**
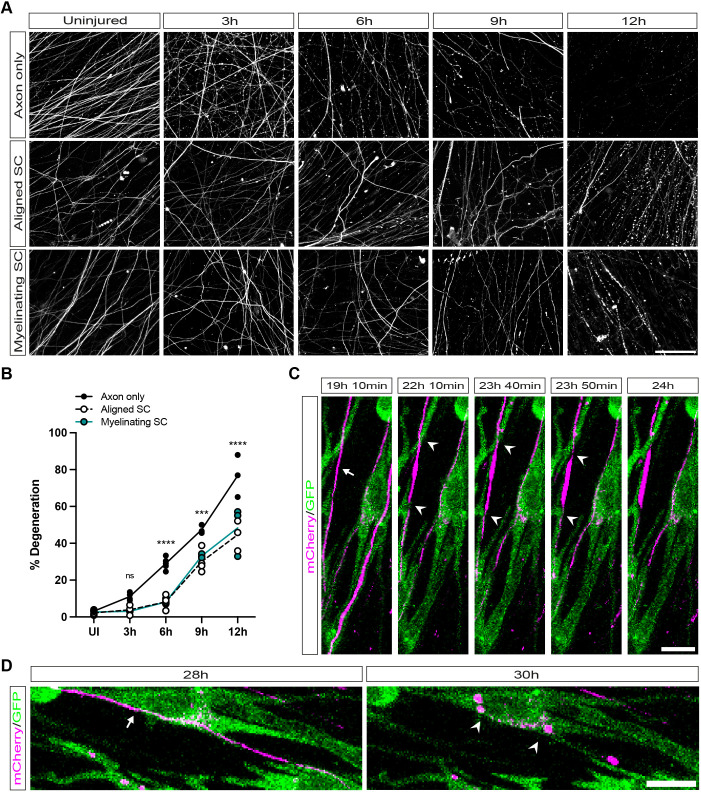
**SCs are axo-protective independently of myelination status at early timepoints, and promote axon fragmentation at later timepoints after axotomy.** (A) Confocal images of NFL-labelled cocultures prior to axotomy at the microgroove barrier (uninjured), and 3, 6, 9 and 12 h after axotomy. Axon-only cultures show earlier signs of degeneration than cultures with SCs. Scale bar: 100 μm. (B) Quantification of axon degeneration after axotomy. Only axon-only cultures show statistically substantial degeneration at 6 h post axotomy (29±1.44%, *n*=5). In axon-only cultures, this degeneration increases to 47.39±1.34% at 9 h post axotomy (*n*=3), and finally 76.68±6.60% at 12 h post axotomy (*n*=3). Cocultures with SCs show little degeneration at 3 h post axotomy (aligned SCs, 4.87±1.65%, *n*=3; myelinating SCs, 3.048±0.06%, *n*=3) and 6 h post axotomy (myelinating SCs, 8.14±0.65%, *n*=4; aligned SCs, 9.19±2.57%, *n*=3). At 9 and 12 h post axotomy, axons associated with both aligned and myelinating SCs start to degenerate (aligned SCs, 9 h, 30.05±3.05%, *n*=4 and 12 h, 44.61±4.72%, *n*=3; myelinating SCs, 9 h, 33.12±0.61%, *n*=3 and 12 h, 48.38±7.75%, *n*=3). There were no significant differences in axon degeneration rates between aligned and myelinating SCs cocultures (3 h, *P*=0.65; 6 h, *P*=0.98; 9 h, *P*=0.44; 12 h, *P*=0.70). Axon-only cultures show significant differences compared to cultures with aligned or myelinating SCs at 6 h (aligned SCs, *P*<0.0001; myelinating SCs, *P*<0.0001), 9 h (aligned SCs, *P*=0.0004; myelinating SC, *P*=0.0059), and 12 h (aligned SCs, *P*<0.0001, myelinating SC: *P*<0.0001) post axotomy. Results shown as individual data points. Statistical significance for comparisons between axon-only cultures and aligned SC cocultures are displayed on the graph. ***P<0.001; ****P<0.0001; ns, not significant. All values are mean±s.e.m.; *n* number refers to independently prepared cultures from separate litters of mice. Statistical analysis was performed with two-way ANOVA with post-hoc Tukey test to correct for multiple comparisons. (C) Confocal images of mCherry-labelled axons (magenta) and GFP-labelled SCs (green). At an early timepoint (19 h 10 min) an intact axon is visible (white arrow); 3 h later (22 h 10 min), the axon is starting to be constricted (white arrowheads). This continues until 23 h 50 min after axotomy, when constrictions are clearly visible, and the axon is swollen between two constrictions. At 24 h after axotomy, the axon then breaks apart. Scale bar: 10 μm. (D) Confocal images of mCherry-labelled axons (magenta) and GFP-labelled SCs (green) with intact axons (white arrow, 28 h post axotomy) just before axon degeneration and with mCherry fragments (white arrowheads) within SCs 30 h post axotomy, once axon degeneration has occurred. Scale bar: 10 μm. All images representative of at least three experimental repeats for studies of degeneration rates (A,B) and two experimental repeats for live imaging of axon fragmentation (C,D).

Recent evidence has shown both axo-protective and axon debris clearance roles for SCs in cocultures. Babetto et al. (2020) used axotomy in rat SCs seeded on mouse DRG axons without inducing myelination, whereas Vaquié et al. (2019) studied laser axotomy in myelinating rat SC–DRG cocultures (Babetto et al., 2020; Vaquié et al., 2019). One outstanding question resulting from both studies is whether myelination status influences the axon degeneration rate *in vitro*. Therefore, we set out to use our cocultures to replicate the findings of both studies in a coculture model made purely from mouse cells and also to investigate whether the myelination status of SCs influences the rate of axon degeneration. We performed axotomies on axon-only cultures, cultures with aligned SCs and cultures with myelinating SCs. Cocultures were fixed at 3, 6, 9 and 12 h post axotomy, and were immunolabelled with NFL to assay axonal integrity (Fig. 4A). Importantly, we found that fixed myelinated cultures needed to be permeabilised with acetone to allow full penetration of NFL antibodies to label axons in their entirety through myelinated segments (data not shown). Although there was negligible degeneration in all cultures at 3 h post axotomy, there was noticeably more degeneration at 6 h post axotomy in axon-only cultures (29.00±1.64%, *n*=5), compared to both aligned SC, and myelinating SC cocultures (8.19±2.57%, *n*=3 *P*<0.0001, and 8.14±0.65%, *n*=4 *P*<0.0001, respectively). Both aligned and myelinating SC cocultures also showed significantly lower amounts of degeneration at both nine and 12 h post axotomy in comparison to axon-only cultures (axon only: 9 h, 47.39±1.34%, *n*=3, and 12 h, 76.68±6.60%, *n*=3; aligned SC: 9 h, 30.05±3.05%, *n*=4, *P*=0.0004 and 12 h, 44.61±4.72%, *n*=3, *P*<0.0001; myelinating SC: 9 h, 33.12±0.61%, *n*=3, *P*=0.0059 and 12 h, 48.38±7.75%, *n*=3, *P*<0.0001; all results mean±s.e.m.; Fig. 4B). Between aligned and myelinating SC cocultures, there were no significant differences in amounts of axon degeneration (3 h, *P*=0.65; 6 h, *P*=0.98; 9 h, *P*=0.44; 12 h, *P*=0.70).

Given that Vaquié et al. (2019) showed SCs appear to accelerate axon degeneration in rat cocultures at later timepoints after axotomy, we were interested to see whether SCs in our cocultures help break up, ingest and clear axonal fragments. We used cocultures where axons were transduced with LV-CMV-mCherry and SCs with LV-CMV-GFP and we live imaged cultures for up to 48 h post axotomy. In these cocultures, we visualised axons break into large fragments surrounded by GFP-positive SC processes, similar to the constricting actin spheres described by Vaquié et al. (2019). We then visualised SCs phagocytose and digest mCherry-labelled axonal fragments (Fig. 4C,D; Movie 1). When we quantified this phenomenon, we found that 97.84% (mean, *n*=2) of SCs in our cocultures contained mCherry-labelled axonal fragments.

Thus, in a dissociated mouse coculture system, the presence of SCs delays the onset of axon degeneration at sites distal from the axotomy. Furthermore, this delay in degeneration does not appear to be reliant on the myelination status of the SCs. At later timepoints, once axons start to degenerate, SCs fragment, ingest and clear axonal debris.

## DISCUSSION

Here, we describe a protocol to set up dissociated mouse myelinating SC–DRG neuron cocultures in microfluidic chambers. These can be utilised to study myelination and injury responses, and can be adapted for the application of drugs to different cellular compartments and for live imaging. Furthermore, as this is a dissociated and compartmentalised purely mouse cell culture system, one can utilise the vast array of transgenic and knockout lines available to study neuron–SC interactions in more detail, without the concern of contaminating endogenous SCs and other non-neuronal cells, which remains a drawback of current mouse dissociated or non-dissociated DRG explant models. Our cultures display several hallmarks of mature myelin sheaths, with electron-dense myelin, compact myelin proteins and a CASPR immunolabelling patterning suggesting assembly of paranodal and nodal structures. Furthermore, we show comparable levels of myelination to that in dissociated rat SC–DRG coculture models. Axotomy of our cocultures faithfully replicates several of the key cellular events seen after nerve injury, including axon degeneration, upregulation of the key injury transcription factor JUN in both aligned and myelinating SCs, and SC demyelination with myelin ovoid formation.

Achieving myelin differentiation of mouse SCs, whether in monocultures or in coculture, has historically been very difficult. There are very few published reports of robust myelination in dissociated mouse SC–DRG neuron cocultures or of myelin differentiation in mouse monocultures (Arthur-Farraj et al., 2011; Stevens et al., 1998). Furthermore, there are no detailed published protocols for either. The majority of the field use rat SCs for *in vitro* myelination studies as it is much easier to achieve myelin differentiation and there are a number of published protocols (Eldridge et al., 1987; Monje, 2020; Morgan et al., 1991; Taveggia and Bolino, 2018; Vaquié et al., 2018). Our protocol differs somewhat from the one used by Stevens et al. (1998) to induce myelination in dissociated mouse SC–DRG cocultures, as they used ascorbic acid and 10% HS and presumably plated their cultures on laminin, though they do not explicitly detail this (Stevens et al., 1998). In our preliminary experiments, we were unable to visualise much myelination with use of laminin, ascorbic acid or indeed if βNRG1 and high concentration forskolin was added to the medium for up to 4 weeks. However, if we plated cocultures on Matrigel^®^ and continuously added it to the myelination medium, then we saw comparable levels of myelination in our mouse cocultures to that of rat cocultures (Eldridge et al., 1987). This approach of using Matrigel^®^ to enhance myelination has previously been successfully employed in cultures of human induced pluripotent stem cell (iPSC) sensory neurons with rat SCs and in non-dissociated mouse DRG explant cultures (Clark et al., 2017; Päiväläinen et al., 2008). Importantly, we used growth factor-depleted Matrigel^®^ as standard Matrigel^®^ preparations contain substantial amounts of transforming growth factor β (TGFβ), which is a known inhibitor of myelination (Einheber et al., 1995). Additionally, the majority of rat and mouse coculture protocols plate cells on glass, whereas we found cultures were healthier and myelinated better when cultured on plastic Alcar^®^ coverslips. Using our protocol, it is possible to set up dissociated compartmentalised mouse myelinating SC–DRG neuron cocultures and take advantage of the ability to use cells from various transgenic mouse lines to study axon–SC interactions during myelination, injury and regeneration. Furthermore, our protocol is complementary to the recently described 3D mouse myelinating SC–motor neuron coculture system using collagen hydrogels (Hyung et al., 2021; Park et al., 2021). It will be interesting in the future to increase the concentration of Matrigel^®^, which is similar to collagen hydrogels, in our cultures to see whether further increasing extracellular matrix viscosity and stiffness improves our myelination efficiency even further. Although it is possible to study cell migration in microfluidic cell culture devices, Transwell models offer significant advantages to study this cellular phenomenon (Negro et al., 2022). To date, there have been no published studies of successful myelination in human SC–neuron coculture systems. Despite this, rat SCs have been shown to readily myelinate human iPSC-derived sensory neurons and an iPSC-derived peripheral nerve organoid system that does contain myelinating SCs has recently been described (Clark et al., 2017; Van Lent et al., 2023).

We used our cocultures to confirm the recently published findings using rat SCs showing that the presence of SCs can delay the onset of axon degeneration and that SCs are able to clear axonal debris after degeneration is initiated. We identified two distinct phases of SC–axonal interaction post axotomy in coculture. At timepoints up to 12 h post axotomy, we observed that SCs delay the initiation of axon degeneration; however, once the axon degenerates, live imaging up to 48 h post axotomy demonstrates that SCs help break axons into large fragments and then ingest and clear axonal debris. These findings confirm the observations of both Babetto et al. (2020) and Vaquié et al. (2019) who used rat SCs in similar microfluidic culture systems (Babetto et al., 2020; Vaquié et al., 2019). We have shown that the axo-protective observation seen by Babetto et al. (2020) does not rely on myelination status, which was an outstanding question from that study. Furthermore, in an advance from previous studies, we have visualised the axo-protective and axon clearance phenomena in the same culture and shown that they are temporally separated, with axon fragmentation and debris clearance by SCs occurring at much later timepoints after axotomy. Several different culture and experimental conditions preclude direct comparison of our study with both those of Vaquié et al. (2019) and Babetto et al. (2020). These include the use of rat SCs in both prior studies and that Babetto et al. (2020) mixed rat SC with mouse DRG axons, as well as length of time in culture, time points quantified after injury and distance from injury and site of analysis. Babetto et al. (2020) performed axotomy on relatively short term cocultures (6 days *in vitro*) whereas Vaiquié et al. (2019) cultured for at least 4 weeks and, in our case, we cultured 6 weeks prior to axotomy. Vaiquié et al. (2019) removed nerve growth factor (NGF) prior to laser axotomy and quantified proximally (although they also imaged distally) whereas both our study and Babetto et al. (2020) kept NGF in the medium, performed axotomy with a scalpel, and quantified more distally and, in our case, extremely distally, where only individual neurites and no axon bundles were visible. Vaquié et al. (2019) had SCs on both sides of the barrier in the microfluidic chambers, whereas we seeded SCs only in the axonal/bottom compartment. Additionally, our cocultures had both forskolin and βNRG1 added to help induce myelination, whereas these factors are not required in rat myelinating cocultures. Finally, it is important to permeabilise myelinated cultures with acetone after fixation, as we did, to visualise the entire axon through heavily myelinated segments, otherwise axon integrity cannot be reliably assessed in a quantitative manner (Babetto et al., 2020; Vaquié et al., 2019).

Multiple independent findings from *in vivo* studies have demonstrated that SCs help break up the axon during or slightly after programmed axonal degeneration is initiated during nerve trauma (Catenaccio et al., 2017; Rosenberg et al., 2014; Vaquié et al., 2019; Villegas et al., 2012). We have also confirmed this phenomenon in a two-photon axotomy model in zebrafish larvae (P. A.-F, unpublished observation). More recently Babetto et al. (2020) showed that SCs upregulate glycolysis within the first 2 days after traumatic nerve injury and this has an axo-protective effect *in vivo* (Babetto et al., 2020). SCs have been shown to promote axonal and neuronal survival in other situations, including during axon regeneration in both the acute and chronic setting, old age and in neuropathy (Arthur-Farraj et al., 2012; Fontana et al., 2012; Hantke et al., 2014; Painter et al., 2014; Wagstaff et al., 2021). It is thus likely that SCs have both axo-protective and axon fragmentation roles *in vivo* after traumatic nerve injury. Future studies will be needed to detail the precise *in vivo* timing of these different cellular phases of SCs on axon integrity after nerve trauma.

One limitation of our coculture model, and indeed most coculture and cell culture models that are used to investigate cellular and molecular mechanisms in nerve injury, is that the cells are obtained from embryonic or neonatal animals. This is an important caveat when applying results from cell culture to adult *in vivo* nerve injury. However, although we would argue that cell culture approaches should always be used in combination with *in vivo* study, it is important to remember that nerve injury is not restricted to adults, and brachial plexus injury secondary to birth trauma is unfortunately a significant clinical problem (Pondaag et al., 2007). Furthermore, neonatal SCs replicate many of key cellular and molecular mechanisms seen in adult SCs after injury, including JUN upregulation, myelinophagy, promotion of axon growth and expression of key repair programme transcripts (Arthur-Farraj et al., 2012, 2017; Gomez-Sanchez et al., 2015; Parkinson et al., 2008). A future development would be to try to adapt this protocol to make a coculture model with adult mouse or even human cells.

In summary, we have described a detailed method of setting up dissociated mouse myelinating SC–DRG neuron cocultures in microfluidic chambers. These cultures can be lentivirally transduced, readily live imaged, used for studying myelination and cellular responses to injury and regeneration, and used for drug studies. Most importantly SCs and DRG neurons from various transgenic mice can be used to perform *in vitro* analysis to complement findings from *in vivo* transgenic mouse studies.

## MATERIALS AND METHODS

### Animals

All research undertaken with animals was performed according to the Scientific Procedures Act 1986 and subject to approval by the University of Cambridge Animal Welfare and Ethical Review Body (AWERB). Wild-type C57BL/6J mice were obtained from Charles River Laboratories and were held under standard specific pathogen-free conditions.

### Immunocytochemistry

Cells were fixed for 10 min in 4% paraformaldehyde (PFA; Electron Microscopy Sciences) diluted in phosphate buffered saline (PBS) at room temperature (RT). Axon-only cultures related to Fig. 1 were permeabilised in PBS plus 0.5% Triton X-100 (Merck), 5% HS (Thermo Fisher Scientific, 16050130) and 5% donkey serum (DS; Merck, D9663) at RT for 1 h. For the purposes of quantifying the rate of axon degeneration (Fig. 4) both axon-only cultures and cocultures with SCs were permeabilised in 50% acetone for 2 min, 100% acetone for 2 min, 50% acetone for 2 min (all at RT), and then blocked in PBS plus 0.5% Triton X-100, 5% HS and 5% DS at RT for 1 h. Myelinating cocultures stained for MBP, MPZ or CASPR were permeabilised in 50% Acetone for 2 min, 100% acetone for 2 min, 50% acetone for 2 min (all at RT), 100% methanol at −20°C for 10 min, and then blocked in PBS plus 5% HS and 5% DS at RT for 1 h. Cultures were immunolabelled by incubating overnight at 4°C with primary antibodies. Primary antibodies were visualised using Alexa Fluor 488-, 568- and 647-conjugated secondary antibodies. DAPI (Thermo Fisher Scientific, D1306) was used 1:10,000. Cells were mounted using Citifluor Glycerol Pbs Solution AF1 (Agar Scientific Ltd) and sealed using nail varnish. For confocal imaging a Zeiss LSM700 or LSM900 with airyscan 2 were used. Images were then processed using Fiji (Schindelin et al., 2012).

### Antibodies

Primary antibodies used were against: CASPR (1:500, Antibodies Inc., 75-001, RRID: AB_2083496), JUN (1:500, Cell Signalling Technology, 9165, RRID:AB_2130165), MBP (EMD Millipore, 1:1000, AB9348, RRID: AB_2140366), MPZ (1:500, Aves Labs, PZO, RRID: AB_2313561), NFL (1:500, Abcam, ab72997, RRID: AB_1267598), PRX (1:500, a kind gift from Peter Brophy, Centre for Discovery Brain Sciences, University of Edinburgh, UK) and SOX10 (1:100, R&D Systems, AF2864, RRID:AB_442208).

Secondary antibodies used were against: donkey anti-goat IgG (H+L) Alexa Fluor 488 (1:500, Thermo Fisher Scientific, A11057, RRID:AB_2534102), goat anti-mouse IgG (H+L) Alexa Fluor 488 (1:500, Thermo Fisher Scientific, A11001, RRID:AB_2534069), goat anti-mouse IgG (H+L) Alexa Fluor 647 (1:500, Thermo Fisher Scientific, A-21235, RRID:AB_2535804), goat anti-rabbit IgG (H+L) Alexa Fluor 568 (1:500, Thermo Fisher Scientific, A11011, RRID:AB_143157), donkey anti-chicken IgY (H+L) Alexa Fluor 488 (1:500, Jackson ImmunoResearch, 703-545-155, RRID:AB_2340375), donkey anti-chicken IgY (H+L) Alexa Fluor 647 (1:500, Thermo Fisher Scientific, A78952, RRID:AB_2921074).

### Quantification of myelination in cocultures

To quantify the number of myelin segments per area, we counted the number of myelin segments for five areas per culture for three cultures and normalised this per mm^2^. To quantify the percentage of SCs in myelinating cocultures that are actively myelinating, we quantified the number of myelin segments and the number of DAPI-positive nuclei for five areas per culture for three cultures. To measure interperiodic distance, we measured at least ten periods per myelinated fibre for at least three fibres per sample for three separate samples.

### Coculture axotomy

All cultures (axon only, aligned SCs and myelinating SCs) were cultured for 6 weeks prior to axotomy experiments. To minimise the possibility that medium constituents were responsible for differences in axon degeneration rates, axonal compartments of axon-only cultures were cultured in medium containing 10 ng ml^−1^ βNRG1 and 10 μM forskolin (see section ‘Axon only medium’ in the step-by-step protocol below) once SCs were seeded onto other cultures, and then switched into myelination medium (additional Matrigel^®^ and 50 μg ml^−1^ L-ascorbic acid), 24 h before axotomy (Fig. S1). Bottom compartments of aligned SC cultures, 24 h before axotomy, were switched into DRG/SC medium containing 10 ng ml^−1^ βNRG-1, 10 μM forskolin and 50 μg ml^−1^ L-ascorbic acid, which is insufficient to induce myelination in mouse cultures. Bottom compartments of myelinating cocultures were medium changed into fresh myelination medium (see section ‘Myelination medium’ in the step-by-step protocol below) 24 h prior to axotomy. Traumatic axotomies were carried out by carefully removing the microfluidic chamber (SND150 and RND150, Xona Microfluidics^®^) from the Aclar^®^ coverslip using sterile forceps and severing axons with a surgical blade under a light microscope. Axotomies were carried out at the level of the microgroove barrier. To confirm all axons were severed, a second higher cut was performed and axons between the cut sites removed using the surgical blade. Vacor axotomies were carried out by medium changing the top compartment to medium supplemented with 50 μM vacor (Greyhound Chromatography, N-13738).

### Quantification of degeneration

Five images at a distance of between 1.2–1.4 mm from the microgroove barrier (the most distal part of the culture that could be imaged) were quantified per culture, taken in comparable locations in each culture. A line was drawn across each image, and each axon crossing this line was either scored as degenerated or intact. Images were anonymised prior to quantification. A minimum of three cultures were assessed per timepoint for each condition.

### Electron microscopy

After removal of the microfluidic chamber, the orientation of the coverslips was marked, and cells were fixed overnight at 4°C in 2% glutaraldehyde and 2% formaldehyde in 0.05 M sodium cacodylate buffer pH 7.4 containing 2 mM calcium chloride. After washing five times with 0.05 M sodium cacodylate buffer pH 7.4, samples were osmicated (1% osmium tetroxide, 1.5% potassium ferricyanide, 0.05 M sodium cacodylate buffer pH 7.4) for 3 days at 4°C. Samples were washed five times in deionised water (DIW) and treated with 0.1% thiocarbohydrazide in DIW for 20 min at RT in the dark. After five further washes in DIW, samples were osmicated a second time for 1 h at RT (2% osmium tetroxide in DIW). After washing five times in DIW, samples were block stained with uranyl acetate (2% uranyl acetate in 0.05 M maleate buffer, pH 5.5) for 3 days at 4°C. Samples were washed five times in DIW and then dehydrated in a graded series of ethanol (50%, 70%, 95%, 100% and 100% dry) and 100% dry acetonitrile, three times in each for at least 5 min. Samples were infiltrated with a 50:50 mixture of 100% dry acetonitrile/Quetol resin (12 g Quetol 651, 15.7 g NSA, 5.7 g MNA, all from TAAB) overnight, followed by 5 days in 100% Quetol resin with 0.5 g BDMA (TAAB), exchanging the resin each day. Aclar^®^ coverslips were placed on top of round polyethylene cups, with cells facing the resin. Samples were cured at 60°C for 3 days, and coverslips removed. The required section plane was marked on the block, and smaller sample blocks were cut from the resin using a hacksaw and mounted on resin stubs. Thin sections (∼ 70 nm) were prepared using an ultramicrotome (Leica Ultracut E) and collected on bare Cu TEM grids or Cu/carbon film grids. Samples were imaged in a Tecnai G2 TEM (FEI/Thermo Fisher Scientific) run at 200 keV using a 20 μm objective aperture to improve contrast. Images were acquired using an ORCA HR high resolution CCD camera (Advanced Microscopy Techniques Corp, Danvers USA).

### Live imaging of cocultures

Microfluidic chambers were placed on a Zeiss LSM 900 confocal microscope equipped with a temperature-controlled chamber at 37°C 5% CO_2_. Multiple areas of interest were selected for each microfluidic chamber and imaged every 10 min for up to 48 h. To quantify number of SCs with fragments, each cell was defined as a region of interest and checked for the presence of mCherry-positive fragments at all timepoints. Two separate independently prepared cultures and cells in ten areas per culture were analysed.

### Statistical analysis

Results are shown as mean±s.e.m. or as violin plots with the median and the upper and lower quartiles marked. Statistical significance was estimated by a two-tailed unpaired Student's *t*-test or two-way ANOVA with a post-hoc Tukey test to correct for multiple comparisons. *P*<0.05 was considered statistically significant. Statistical analysis was performed using GraphPad Prism software (version 9.5.0).

### Step-by-step dissociated mouse myelinating SC–DRG compartmentalised coculture protocol

#### Dissociated embryonic mouse DRG neuron culture

##### Aclar^®^ plastic coverslip preparation

- Aclar^®^ coverslips were obtained from Electron microscopy sciences (50425-10). Process by:
- Cut into 40×22 mm pieces.
- Autoclave in glass dish.

##### Day 1

Preparing the dishes:

- Make a 750 μl drop of 0.5 mg ml^−1^ PLL (Merck, P1274) in a dish and place one Aclar^®^ coverslip on this drop, using sterilised forceps (kept in 70–100% ethanol for at least 24 h prior to usage).
- Leave to coat overnight at room temperature (RT).

DRG dissection:

- Dissect as many DRGs as possible from E14 mice:
  o Place the embryo in a dish containing ice-cold L15 (Thermo Fisher Scientific, 11415049) in a sterile dissection hood.
  o Lay the embryo on its side and remove head and ventral part of the embryo (carefully remove skin and internal organs, liver and gut particularly, using no. 5 forceps).
  o Remove any remaining tissue in front of the vertebral column, using forceps.
  o Place vertebral column ventral side up and use micro-dissecting scissors or forceps to cut/crush through vertebral column.
  o Open up the vertebral column by gently teasing apart the right and left side to expose the spinal cord and DRGs.
  oFrom the cranial end use a no. 5 forceps to gently remove the spinal cord from the open vertebral column and move it to a 35 mm dish containing hibernate medium (see ‘Media’ section below). All the DRGs should remain attached to the spinal cord.
  o In the 35 mm dish simply remove DRGs one by one using no. 5 forceps. Transfer all DRGs using a P1000 into fresh hibernate medium. Only transfer 50 or so at a time and use just the tip of the P1000 pipette tip as the DRGs will get stuck on the plastic if they are sucked too far up the P1000 tip.
- Store overnight at 4°C.

##### Day 2

Preparing the PLL-coated Aclar® coverslips:

- Remove the PLL and always keep the side of the Aclar® coverslips that is coated with PLL facing up through the washing process below. Using sterile forceps to manipulate the coverslips.
- Wash coverslip with sterile ultrapure H_2_O (resistivity 18.2 MΩ·cm at 25°C).
- Airdry coverslips and move to a new sterile 60 mm dish.
- Place Xona Microfluidics^®^ chambers (either SND150 or RND150), which have a 150 μm microgroove barrier, on top of coverslip using sterile forceps.
- Check chamber attachment under microscope until there are no visible air bubbles between the chamber and the PLL coated Aclar^®^ coverslip.
- Place three 60 mm dishes containing a PLL-coated Aclar^®^ coverslip in an upside down large 200 mm sterile petri dish.

Matrigel^®^ coating:

- Thaw an aliquot of growth factor depleted Matrigel® (Corning, 356231) on ice and dilute 1:200 in cold DMEM high glucose (4500 g dl^−1^ glucose; Thermo Fisher Scientific, 41966029).
- For both top compartments of the microfluidic chamber (see diagram in Fig. 1), pipette 150 μl into the right well making sure it flows through the top channel into the left well: make sure to pipette forcefully in one fluid motion right at the channel entrance to minimise any air bubbles in the channel.
- Remove almost all of the volume of 1:200 Matrigel^®^ in DMEM from both wells, making sure to leave medium in the top channel and pipette into the left well to encourage flow.
- Repeat the steps above for the bottom compartment.
- Leave chambers in their 60 mm dish coating with Matrigel^®^ for at least 1 h at 37°C. They can be placed back inside their upside down large sterile Petri dish for safety.
- Add an open (remove and discard lid) 35 mm sterile dish full of sterile water to the large sterile petri dish to maintain humidity and stop the chambers from drying out.

Dissociation:

- You will need cells from ∼10 DRG/ganglia per chamber for stable long-term cultures.
- Warm up 2.5 ml of 0.025% Trypsin (Merck, T9201) dissolved in Ca^2+^ and Mg^2+^-free PBS (Merck) in a 15 ml falcon in a water bath at 37°C.
- Make up DRG medium (see ‘Media’ section below) and set aside enough DRG medium for dissociation and topping up wells the next day. We generally prepare 2.5 ml DRG medium per 10 ganglia/chamber.
- Transfer ∼40 ganglia into the warm 15 ml falcon containing 2.5 ml of 0.025% trypsin.
- Leave for 30 min at 37°C.
- While DRGs are trypsinising, warm up 600 μl of collagenase solution in a 1.5 ml Eppendorf at 37°C in a water bath. Collagenase solution is 682 U ml^−1^ collagenase (Worthington Biochemical Corporation - LS004176) in Ca^2+-^ and Mg^2+^-free medium [10% Krebs solution (133 mM NaCl, 177 mM KCL, 1.75 mM NaH_2_PO_4_, all Merck), 1% MEM non-essential amino acids solution (Thermo Fisher Scientific), 0.5% Phenol Red solution (Merck), 0.2% NaHCO_3_ (Thermo Fisher Scientific), 0.2% Glucose (Thermo Fisher Scientific)].
- Transfer ganglia into warmed 600 μl of collagenase in a 1.5 ml Eppendorf (transfer only ganglia, not trypsin).
- Mix liquids but not cells.
- Leave for 30 min at 37°C.
- Prepare one 1 ml DRG medium in a 15 ml falcon per Eppendorf of DRGs.
- Transfer each Eppendorf of DRGs to 1 ml DRG medium in a 15 ml falcon.
- Triturate with P1000 pipette very gently – do not over dissociate (this leads to low yields and unhealthy cultures). Move any lumps of tissue left to a separate tube to triturate further, if necessary.
- Centrifuge for 5 min at 260 ***g*** (room temperature, RT).
- For lentiviral infection: resuspend in 1 ml DRG medium (and proceed to next section).
- For plating: resuspend in DRG medium (1 μl per ganglion) and pool tubes (proceed to ‘Plating’ section below).

Lentiviral infection of DRGs:

- Lentiviruses stored at −80°C and thawed on ice (you can re-use lentivirus aliquot once after thawing but use a double volume on the subsequent experiment as viral copies reduce by ∼50% every freeze–thaw cycle in our experience).
- Add virus to DRGs resuspended in 1 ml of DRG medium.
- Use a multiplicity of infection (MOI) of 2−10 for transducing DRGs.
- Leads to ∼100% transduction.
- Centrifuge for 1 h at 30 ***g*** (RT).
- Resuspend pellet in 1 μl per ganglion DRG medium+the same amount of virus added previously.
- Proceed to plating.

Plating:

- Remove 1:200 Matrigel^®^ in DMEM from top and bottom wells of chambers (leaving just medium in the top and bottom channels to avoid air bubbles) and replace with 100 μl of DRG medium per compartment, pipetting in the same way as is described in the ‘Matrigel^®^ coating' section above.
- Immediately afterwards, remove all DRG medium from the top wells (leaving just medium in the channel to avoid air bubbles).
- Load 10 μl cells into the top channel by pipetting gently in one fluid motion right at the channel entrance (either right or left side).
- Check underneath microscope and if cells look sparse, load another 5–10 μl of cells from the other side.
- Allow 4 h for cells to attach.
- Top up with 100 μl added to top compartment: make sure to pipette from well to well to keep volume the same and reduce flow.
- Check that the medium level is higher in the bottom compartment, if not, add one or two drops to establish volume difference.

##### Day 3

Topping up:

- For square chambers (SND150, Xona Microfluidics^®^), top up wells with 200 μl added to the top compartment and 300 μl to the bottom compartment.
- For round chambers (RND150, Xona Microfluidics^®^), top up wells with 75 μl added to the top compartment and 150 μl to the bottom compartment.
- Check that the medium level is higher in the bottom compartment, if not, add one or two drops to establish volume difference.
- Add anti-mitotic agent cytosine arabinoside (Ara-C, Merck, C6645) to the top compartment at final concentration 10^−5^ M. Add half of the total volume to the left and right top wells.

##### Changing medium

- Aim to change medium on Mondays, Wednesdays and Fridays.
- Make sure to pipette one drop per well into alternating wells to reduce flow.
- Make sure to top up the water in the dish to stop chambers drying out.

##### Day 10

- After maintaining DRGs in Ara-C for 7 days, change medium to DRG medium without Ara-C.
- Non-neuronal cells will not return after this stage.
- Make sure to wait for axons to extend to the wells before seeding SCs. If not, then leave cultures for a few more days before seeding SCs. Make sure Ara-C has been removed 3 days prior to SCs being added, and medium has been changed twice, to minimise any possible toxicity (not experienced in our hands).

#### Dissociated neonatal mouse SC culture

- Dissect P3–P5 sciatic nerves and brachial plexuses from mice and start SC culture within 3–5 days of DRG dissociation.
- After 3 days of Ara-C purification, trypsinise cells and infect with lentiviruses in suspension (see below sections).
- Expand SCs on 60 mm PLL/laminin coated dishes until needed for coculture (section ‘Mouse SC expansion’ below).

##### Preparing dishes

PLL coating:

- Prepare a 0.2 mg ml^−1^ solution of PLL and coat 35- or 60-mm sterile dishes overnight.
- Remove PLL (can be re-frozen and used three times).
- Wash three times with sterile ultrapure H_2_O (resistivity 18.2 MΩ·cm at 25°C).
- Leave dishes to air dry, store at RT.

Laminin coating:

- Dilute the stock solution of laminin (Merck, L2020) in low-glucose DMEM (1000 g dl^−1^; Thermo Fisher Scientific, 21885025) to a final concentration of 10 μg ml^−1^ (1:100 dilution).
- Add the solution to the dish.
- Leave for at least 1 h at 37°C.
- Remove laminin immediately prior to plating cells (can be reused three times) and do not let dishes dry (add medium).

##### Mouse SC purification

- Make 20 ml of DMEM low-glucose [with 1:100 penicillin-streptomycin (Thermo Fisher Scientific, 15140122) plus 5% HS (Thermo Fisher Scientific, 16050130)] and warm to 37°C.
- Prepare 2×60 mm tissue culture dishes of L15 and place on ice.
- Dissect out sciatic nerves and brachial plexuses and place in ice-cold L15 (4-6°C).
- De-sheath the nerves and transfer to a separate dish containing L15.
- Place 100 μl trypsin and 100 μl collagenase (per two animals) in a 35 mm dish: 2 mg ml^−1^ trypsin (Merck, 85450C) and 682 U ml^−1^ Collagenase in Ca^2+^- and Mg^2+^-free medium.
- Transfer the nerves into the trypsin/collagenase and incubate at 37°C for 45 min.
- Triturate nerves with a P1000 and then with a P200.
- Stop the digestion by adding an excess 2 ml low glucose DMEM plus 5% HS.
- Transfer the cell suspension to a 15 ml centrifuge tube.
- Centrifuge at 180 ***g*** for 10 min (RT).
- Resuspend the cell pellet in 2 or 4 ml of low glucose DMEM plus 5% HS.
- Remove laminin/DMEM solution from 35- or 60-mm dishes (store at 4°C for up to 1 month and can be re-used maximum of three times).
- Transfer cell suspension to the 35- or 60-mm laminin-coated dishes.
- Add Ara-C to a final concentration of 10^−5^ M and culture for 3 days to eliminate fibroblasts.
- After 3 days, replate SCs and expand (see next section) or lentivirally transduce (see section ‘Lentiviral infection of mouse SCs’ below) prior to expanding.

##### Mouse SC expansion

- Mouse SCs proliferate on PLL/laminin coated dishes in the presence of low serum, βNRG1, and a low concentration cAMP signal (Arthur-Farraj et al., 2011; see section ‘SC expansion medium’ below).
- Laminin coat 60 mm PLL-coated plates (see ‘Laminin coating’ in the ‘Preparing dishes’ section for the dissociated neonatal mouse SC culture above).
- Wash cells twice with PBS at RT.
- Trypsinise Ara-C purified SCs using 1 ml of 6% 2 mg ml^−1^ trypsin (Merck, 85450C) in Versene [0.02% EDTA (Thermo Fisher Scientific, D/0700/53) plus 0.5% Phenol Red in PBS] for up to 5 min at 37°C.
- Stop the reaction by adding 2 ml (or more) of low glucose DMEM plus 5% HS (pre-warmed to 37°C in a water bath).
- Transfer the cell suspension to a sterile 15 ml centrifuge tube.
- Centrifuge at 180 ***g*** for 10 min (RT).
- Pre-plate the cells to eliminate fibroblasts if needed:
  o resuspend pellet in 10 ml of low glucose DMEM plus 5% HS (if first passage) or defined medium (DM, Table S1) with 0.5% HS (if they have already been passaged once).
  o add to an uncoated sterile 90 mm tissue culture dish (no PLL, no laminin) for 2–3 h.
  o fibroblasts will sit down and attach whereas SCs will remain in suspension.
  o collect medium and wash the dish well with a few extra mls of medium.
  o centrifuge at 180 ***g*** for 10 mins at RT.
- Resuspend cell pellet in SC expansion medium (see ‘Media’ section) and plate or lentivirally infect (see next section) prior to expanding.
- Change medium every 3–4 days (e.g. Monday and Thursday).
- Split cells when 80% confluent.
- Do not passage cells more than three times as mouse SC tend to quiesce after this.

##### Lentiviral infection of mouse SCs

- Pre-plate cells (section directly above) and then lentivirally infect, as described below, prior to expanding.
- Resuspend cell pellet in 1 ml of DM plus 0.5% HS in a 15 ml sterile centrifuge tube.
- Calculate the total number of cells using a haemocytometer or other cell counter.
- LVs are stored at −80°C and thawed on ice.
- Add virus (MOI of 200–500) to cell suspension.
- Centrifuge for 1 h at 30 ***g*** at RT. Centrifugation increases the transduction efficiency.
- Resuspend the pellet in the same supernatant so as to allow more LV to infect cells over the next 24 h.
- Plate SCs on laminin-coated PLL 35 mm or 60 mm dishes (see laminin coating in the ‘Preparing dishes’ section for the dissociated neonatal mouse SC culture above).
- Change medium after 24 h to fresh SC expansion medium (see ‘Media’ section below).
- Proceed with normal expansion (section directly above).

##### Quiescing SCs

- The day before they are used in an experiment, SC cell cycles are synchronised by quiescing them.
- Change media to DM plus 0.5% HS **without** any forskolin or neuregulin.

#### Dissociated mouse DRG neuron–SC coculture

##### Seeding SCs

- Change medium for both wells in the top compartment (DRG cells side) into DRG/SC medium (see ‘Media’ section below).
- There are ∼400,000–600,000 SCs in an 80–90% confluent 60 mm dish.
- Trypsinise SCs with 1 ml of 6% 2 mg ml^−1^ trypsin in Versene for a maximum of 5 min at 37°C.
- Stop the reaction with DMEM low glucose plus 5% HS.
- Centrifuge for 10 min at 180 ***g*** at RT.
- Resuspend in DRG/SC medium to achieve a concentration of 30,000 cells per 10 μl media (3,000,000 ml^−1^).
- Remove almost all the medium from bottom compartment (Fig. 1A), remembering to leave medium in the bottom channel, so as to avoid air bubbles.
- Load 30,000 cells by pipetting at the right or left entrance of the bottom channel (Fig. 1A).
- Check underneath microscope and if cells look sparse, load another 5–10 μl of cells from the opposite side of the bottom channel.
- After 4 h, top up with DRG/SC medium to normal levels (see ‘Topping up’ in the ‘Dissociated embryonic mouse DRG neuron culture’ section).
- Change medium on Mondays, Wednesdays and Fridays.

##### Inducing myelination

- Allow 7 days for SCs to align and proliferate before inducing myelination (reducing this time will compromise the myelination).
- Keep top compartment in DRG/SC medium.
- Change bottom compartment to axon-only medium, keep in DRG/SC medium (for aligned SC), or myelination medium (media are as given below).
- Change medium on Mondays, Wednesdays, and Fridays.

#### Media

##### Supplement stocks

- Nerve growth factor (NGF): 100 μg ml^−1^, Thermo Fisher Mouse NGF 2.5S Native Protein (13257019).
- Forskolin: 10 mM in ethanol, Merck, Coleus forskohlii, CAS 66575-29-9, Calbiochem.
- Neuregulin (βNRG1): 10 μg ml^−1^ in in PBS 1% BSA, Recombinant Human NRG1-β1/HRG1-β1 EGF Domain Protein, RD Systems, 396-HB-050.
- Growth factor-depleted Matrigel^®^ (Corning - 356231). Thaw 10 ml in fridge (4–6°C) overnight. Aliquot into 10 μl and 100 μl aliquots, making sure to keep the Matrigel^®^ at 4–6°C at all times (it polymerises above 10°C). We keep our pipette tips in the freezer beforehand and aliquot on ice. Aliquots can then be stored at −20°C.

##### Hibernate DRG medium

- For 50 ml of medium:
  o 48 ml Hibernate E (Thermo Fisher Scientific, A1247601)
  o 1 ml B27 (2%; Thermo Fisher Scientific, 17504044)
  o 500 μl penicillin-streptomycin
  o 500 μl L-glutamine (Thermo Fisher Scientific, 25030081)
- On day of use add NGF to a final concentration of 33 ng ml^−1^ (1:3000).

##### DRG medium

- For 50 ml of of medium: (store at 4°C for up to 4 weeks):
  o 48.5 ml DMEM (high glucose)
  o 1 ml B27
  o 500 μl penicillin-streptomycin
- On day of use add NGF at 1:3000 (final concentration 33 ng ml^−1^).

##### DRG/SC medium

- For 50 ml of medium (store at 4°C for up to 4 weeks):
  o 24.5 ml DMEM (high glucose)
  o 24.5 ml DM (Table S1)
  o 1 ml B27
  o 500 μl penicillin-streptomycin
- On day of use add NGF at 1:3000 (final concentration 33 ng ml^−1^).

##### Defined medium

- DM is according to Jessen et al. (1994); see Table S1 and store at 4°C for up to 4 weeks.

##### Axon only medium

- For 50 ml of medium (store at 4°C for up to 4 weeks):
  o 24.5 ml DMEM (high glucose)
  o 24.5 ml DM (Table S1)
  o 1 ml B27
  o 500 μl penicillin-streptomycin
- On day of use add:
  o NGF 1:3000 (final concentration 33 ng ml^−1^).
  o Forskolin 1:1000 (final concentration 10 μM).
  o βNRG1 1:1000 (final concentration of 10 ng ml^−1^).

##### Myelination medium

- For 50 ml of media (store at 4°C for up to 4 weeks):
  o 24.5 ml DMEM (high glucose)
  o 24.5 ml DM (Table S1)
  o 1 ml B27
  o 500 μl penicillin-streptomycin
- On day of use add:
  o Matrigel^®^ 1:100 (you can make a 5- or 10-ml stock with Matrigel^®^ added)
  o NGF 1:3000 (final concentration 33 ng ml^−1^)
  o Forskolin 1:1000 (final concentration 10 μM)
  o βNRG1 1:1000 (final concentration 10 ng ml^−1^)

To wells, also add 50 μg ml^−1^ L-ascorbic acid (1:100 from stock; Merck, A4544), make up stock (in H_2_O) fresh each time and protect from light.

##### Schwann cell expansion medium

- Make on day of use.
- For 50 ml of media:
  o 50 ml DM (Table S1)
  o 0.5% HS
  o 10 ng ml^−1^ βNRG1 (R&D Systems, 396-HB-050)
  o 2 μM forskolin (Merck, 344270) or 50-100 μM dibutryl-cAMP (dbcAMP; Merck, D0627).

## Supplementary Material

10.1242/joces.261557_sup1 Supplementary information
