# Supplementary material for: Schwann cells are axo-protective after injury irrespective of myelination status in mouse Schwann cell–neuron cocultures
Source: J Cell Sci. 2023 Sep 20;136(18):jcs261557. doi: 10.1242/jcs.261557 (PMC10546878; doi:10.1242/jcs.261557)
Supplement: Supplementary information [file joces-136-261557-s1.pdf]

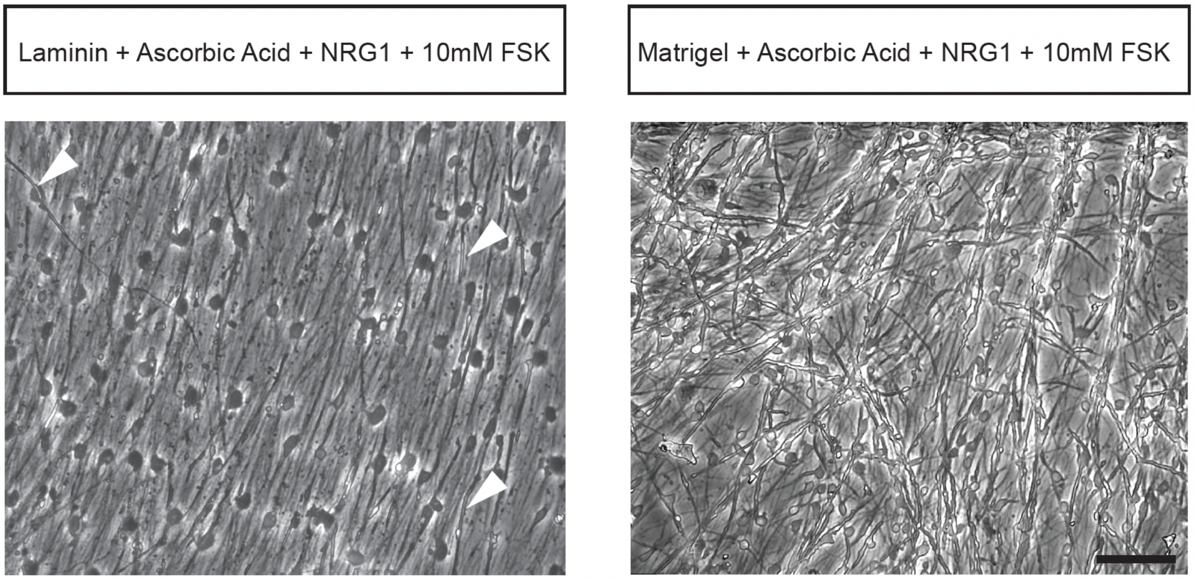

Fig. S1. Ascorbic Acid, neuregulin and elevation of cAMP are insufficient to induce substantial myelination in mouse SC-DRG dissociated cocultures without Matrigel®. The left panel shows a phase image of the bottom channel from cocultures cultured on laminin and treated with ascorbic acid (50 μg ml^{-1}), βNRG1 (10 ng ml^{-1}) and forskolin (10 μM) for four weeks. White arrowheads identify small infrequent myelin sheaths. The right panel shows a phase image of the bottom channel from cocultures cultured on growth factor reduced Matrigel® and treated with ascorbic acid (50 μg ml^{-1}), βNRG1 (10 ng ml^{-1}) and forskolin (10 μM) and 1/100 Matrigel® for four weeks. Extensive myelination with long myelin sheaths is visualised. Scale bar 100 μm.

Journal of Cell Science • Supplementary information

Table S1. Defined medium components.

	Reagent	Source	Final Concentration
50 ml	Hams F12	Thermo Fisher - 31765035	47%
50 ml	DMEM (low glucose)	Thermo Fisher - 21885025	47%
2 ml	L-Glutamine	Merck - 25030081	2 mM
1 ml	Penicillin Streptomycin	Thermo Fisher	100 μM
1 ml	3.5% Bovine Serum Albumin	Merck - A7979	0.035%
1 ml	1.6 mg ml^{-1} Putrescine	Merck - P5780	16 μg ml^{-1}
1 ml	10 mg ml^{-1} Transferrin	Merck - T1283	100 μg ml^{-1}
100 μl	400 μg ml^{-1} L-Thyroxine (T4)	Merck – T1775	400 ng ml^{-1}
100 μl	0.06 mg ml^{-1} Progesterone	Merck - P0130	60 ng ml^{-1}
100 μl	5.7 mg ml^{-1} Insulin	Merck - I9278	10^{-6} M
77 μl	0.05 mg ml^{-1} Dexamethasone	Merck - D4902	38 ng ml^{-1}
10 μl	0.101 mg ml^{-1} L-Thyronine (T3)	Merck - T6397	10.1 ng ml^{-1}
10 μl	1.6 mg ml^{-1} Selenium	Merck - S5261	160 ng ml^{-1}

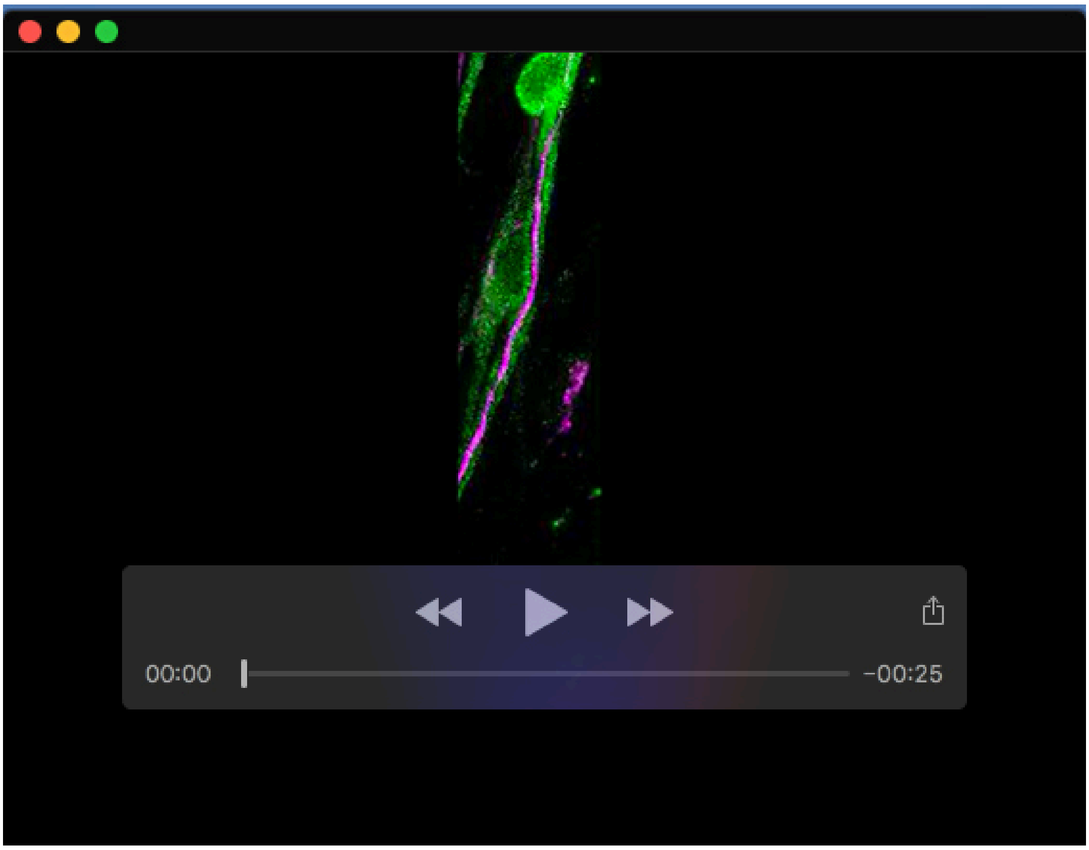

Movie 1. Axonal fragments are present in Schwann cells after axotomy. 48-hour confocal live imaging of mCherry-labelled axons (magenta) and GFP-labelled SCs (green). Initially, intact axons are present, that then swell and break into large pieces, surrounded by SC processes resembling the constricting actin spheres shown by Vaquié et al., 2019. Larger axonal fragments are broken into smaller pieces and are transported along SC projections towards the perinuclear cytoplasm. Cultures were imaged every 10 minutes for 48 hours. 12 frames per second.
