# Supplementary material for: Schwann cells are axo-protective after injury irrespective of myelination status in mouse Schwann cell–neuron cocultures
Source: J Cell Sci. 2023 Sep 20;136(18):jcs261557. doi: 10.1242/jcs.261557 (PMC10546878; doi:10.1242/jcs.261557)
Supplement: Supplementary file 1 [file joces-136-261557_review_history.pdf]

Journal of Cell Science

Review COMMONS

Schwann cells are axo-protective after injury irrespective of myelination status in mouse Schwann cell-neuron cocultures

Clara Mutschler, Shaline V. Fazal, Nathalie Schumacher, Andrea Loreto, Michael P. Coleman and Peter Arthur-Farraj
DOI: 10.1242/jcs.261557

Editor: Giampietro Schiavo

Review timeline

Submission to Review Commons:	24 May 2023
Submission to Journal of Cell Science:	14 August 2023
Editorial decision:	16 August 2023
First revision received:	17 August 2023
Accepted:	18 August 2023

Reviewer 1

Evidence, reproducibility and clarity

Summary:

In this original article from Mutscher et al., the authors developed a compartmentalized dissociated mouse myelinating SC-DRG coculture system to investigate the distinct roles of Schwann cells in axon protection and degeneration after injury. The innovation of this approach relies on (i) the use of mouse SCs and mouse DRGS neurons instead of rat cells; (ii) the use of microfluidic chambers, seeded by axons and SCs in different compartment; (iii) the possibility to perform a traumatic injury in vitro. While this novel approach offers new ways to study peripheral nerve regeneration and SC-axon interaction, and technical the study is robust, the paper is currently limited by the exploration of their model.

Major points:

It is unclear is this approach will ever lead to the identification of key mechanism or key candidates. This is a major miss in the current manuscript form. In short: the authors should demonstrate that their in vitro system can lead to significant leap in our understanding of peripheral nerve regeneration by identifying novel targets/pathways or mechanisms.

The use of embryonic DRG neurons or SC isolated from P2 animals are arguably physiologically not the same cells that are affected by traumatic nerve injury, which happen most often than not in adult. This is a problem in the long-term reliance on this approach to study axotomy peripheral nerve regeneration.

Moderate points:

"there are no established protocols in the field describing the use of mouse SCs with mouse DRG neurons in dissociated myelinating cocultures". The use of mouse cells is laudable, but it is not necessarily a technical innovation, or at least the current manuscript does not explain why their approach particularly suitable to mouse Schwann cells.

The figures in the paper are largely descriptive. They are very little quantitative measurement. Thus, the readers will have a hard to determine, if they replicate the proposed approach, whether their efficient is on par with the current authors.

© 2023. Published by The Company of Biologists under the terms of the Creative Commons Attribution License (https://creativecommons.org/licenses/by/4.0/).

Minor points:
Fig.4B and E should show individual data points.

Significance

In addition, to the demonstration of feasibility of this in vitro approach, the main finding by the authors is that SCs have a role in neuronal protection and support is key for peripheral nerve regeneration. Thus while in vitro approach does not add key information that do not already exists in the field, it somewhat confirms that the effect is SC autonomous. Overall the approach is interesting and has potential, but the study currently lack a demonstration of its usefulness to the community.

It would have been interesting to have the authors discuss the advantages of their approach in comparison to other innovative approaches to study SC-axon interactions that have been developed in the last decade (i.e., 3D environment, microfluidic approach, transwell systems). There is also a lack of citations about similar studies in the field.

Because of the lack of key novel mechanisms, and lack of discussion on what this approach is superior to others in vitro approach limits the impact of the study and the excitement of the reader, even from the SC-axon community.

Reviewer 2

Evidence, reproducibility and clarity

In the manuscript entitled "Distinct axo-protective and axo-destructive roles for Schwann cells after injury in a novel compartmentalised mouse myelinating coculture system", Arthur- Farraj and colleagues detail a method of dissociated coculture of mouse-DRG neurons and Schwann cells in microfluidic chambers. In this system, neurons and Schwann cells harvested from the same or from different animals are grown in different compartments that are connected by microgrooves, thereby allowing for spatial and diffusive separation. Neurons are shown to extent their axons across the microgroove barrier to the glial compartment where Schwann cells align with the axons and myelination can be induced. Detailed analysis of myelination and axon injury/degradation are presented as use cases, including the capability to genetically and pharmacologically manipulate neurons and Schwann cells independently, which also enabled fluorescent life cell imaging. The authors then examine the effect of immature/premyelinating and myelinating Schwann cells on the rate of axon degeneration. Upon axotomy Schwann cells significantly delayed degeneration, with no difference between non-myelinating and myelinating Schwann cells. Finally, live imaging during axon degeneration with fluorescent proteins separately expressed in neurons and Schwann cells demonstrated that Schwann cells ingest axonal fragments.

Major comment:

In establishing a much needed in-vitro system for PNS myelination and injury research the paper represents a valuable contribution to the PNS community. However, I find the presentation of aspects concerning a protective/destructive role of Schwann cells somewhat inconclusive. That these roles exist has been known, as the authors discuss. Then what does this study contribute concerning the open question that was raised by the discrepancies between Babetto et al., 2020 and Vaquié et al., 2019, i.e. how Schwann cells contribute to axon survival/regeneration after injury? Essentially, the only significant conclusion in this regard is that myelinating and non-myelinating mouse Schwann cells do not differ in their capability to protect axons from degeneration. The manuscript, including the title, would benefit from focusing more on this aspect. In particular, the discussion of the factors that lead to the still remaining apparent discrepancies between Babetto et al., 2020 and Vaquié et al., 2019 and this study should be revised. The authors state that "The study by Vaiquié et al., 2019 quantified axon fragmentation proximally in the microgrooves at timepoints starting from 12 hours after axotomy." (Discussion). While this observation is accurate, Jacob and colleagues also show accelerated, obviously distal

© 2023. Published by The Company of Biologists under the terms of the Creative Commons Attribution License (https://creativecommons.org/licenses/by/4.0/).

axon degeneration in the presence of Schwann cells (Figure 3C in Vaquié et al., 2019). It is therefore unlikely that the discrepancies stem from analysis of more proximal vs. more distal axons, or the timepoints of analyses. In my opinion, a further study (using the coculture system presented in this manuscript) that compares the role of Schwann cells from rats and mice, and that includes analysis of more proximal and distal axon degeneration as well analysis of axon regeneration is needed. In a rework of the manuscript, the authors may therefore elaborate more on the shortcomings of the present study, or alternatively soften the aims of the study in the first place.

Minor comments:

- Figure 2D: From the electron micrograph there is no doubt that compact myelin is formed, however to me it seems the compaction is not complete. A rough estimation with the aid of the provided scale bar resulted in an interperiodic distance of about 17 nm, which contrasts with the remarkably well reproduced values reported in multiple reports using conventional specimen preparation (like in this study), of which I am citing just a few: about 13 nm in rat ex vivo nerve (Peterson and Pease, J. Ultrastruct Res 1972; Fledrich et al., Nat Commun 2018), 12 nm (Giese et al., Cell 1992), 12.2 nm (Perot et al., J Neurosci 2007), about 12 (Fernando et al., Nat Commun 2016), about 13 nm (García-Mateo et al., Glia 2017) or about 13 nm in mouse ex vivo (Boutary et al., Commun Biol 2021), which was also reproduced with about 13 nm in rat in vitro (Taveggia and Bolino, Methods Mol Biol 2018). The authors should acknowledge this deviation and might discuss possible reasons.

- Figure 4A,B: The result of a slowed axon degeneration in coculture relies on the accurate assessment of continuity of the NFL staining. While the authors report that acetone permeabilization was necessary to afford complete penetration of the used antibodies in myelinating cultures, I cannot see why the authors have not used the same staining protocol for all cultures, as it is detailed in the method section. While I consider it unlikely that the staining conditions have led to an apparent delay of degeneration in coculture, experiments should generally be performed under identical conditions, unless there are good reasons not to do so. If this is not the case, it will be reassuring to see the same effect when identical staining conditions are employed. On the same note, do the compared cultures have the same age, i.e. have the neuron monocultures been in vitro for the same time as the cocultures?

- In several instances of the manuscript, the term "transfection" is used to refer to lentiviral gene transfer. I advise to use the more appropriate term "transduction" instead

- I could not seem to find a meaningful reference to the microfluidic chambers that were used in the study. The protocol should contain details on the device and source of supply in order to enable potential readers to execute the protocol

Significance

The paper presents a convincing establishment of a dissociated coculture derived exclusively from mouse that leads to robust myelination. As the manuscript correctly states, Schwann cell culture and especially coculture with neurons has been experienced difficult in the field, and by providing a detailed protocol as well as demonstrating how the coculture system can be used to address important questions of PNS myelination and repair, the paper fills an important gap. However, the experiments directed to the role of Schwann cells in axon degeneration do not clarify much, which should be better addressed in the discussion and also by modifying the title accordingly.

The paper will be of high value for basic researchers that are interested in performing studies addressing cellular and molecular mechanisms of myelination and repair in the PNS. Importantly, the paper can pave the way to usage of transgenic or knockout mouse models in coculture. Thereby it might spark interest also in those researchers that use transgenic and knockout mouse models and who have so far refrained from using coculture models.

© 2023. Published by The Company of Biologists under the terms of the Creative Commons Attribution License (https://creativecommons.org/licenses/by/4.0/).

Field of expertise of the reviewer:* Cellular and molecular mechanisms of myelination and growth signaling in the PNS; in-depth experience with DRG coculture models from rats and mice

Reviewer 3

Evidence, reproducibility and clarity

Summary

The authors present a detailed protocol for co-cultures of mouse DRGs with mouse SCs using microfluidics. In this model, cells of interest grow in different compartments while allowing for axons to grow in between, thereby making them accessible to injury induction. Using this experimental system, the authors show that myelination occurs, myelin gets compacted and acquires nodal organization. The authors then show that such a system allows for compartment-specific lentivirus transduction and live imaging. Next, they perform physical and chemical axonal injury and show that at early time point pos- injury the presence of SCs protects from axonal degeneration regardless of the myelination status, and helps with clearing of damaged axons at later time points.

Major comments:

The novelty of the study is questionable.

While the model is well described and appears to be useful for the proposed applications (live imaging, transduction, injury model), the arguments provided regarding its novelty are not fully convincing. The main argument from the authors of this paper is that there are no established protocols describing the use of mouse SCs with mouse DRG neurons in dissociated myelinating cocultures. However, this appears to be inaccurate, as the model described in Stevens et al., 1998 (cited in the paper) uses mouse DRG neurons dissected at E13.5 with mouse SCs dissected at P3 to study myelination. Also, in Päiväläinen et al., 2008, mouse DRGs and SCs are cultured from transgenic mice at different developmental ages, thereby arguing that coculture models have been previously successfully implemented. The main difference appears to be rather the compartmentalization of SCs and DRGs which appears to be a mouse adaptation of the rat model described by Vaquie et al,2019. Based on the above, it seems imperative for the authors to tone down the novelty aspect and provide a more thorough discussion on how the current novel differs from protocols in published study, highlighting advantages and caveats for each.

Next, the authors emphasize the conflicting results of two articles, Babetto et al., 2020 and Vaquie et al., 2019, as the basis to use their newly developed model in the same species and testing two ages corresponding to distinct myelination states. However, both studies reach the same conclusion as the current study, that SCs have a protective role, although at two different developmental time points. As such, it is likely that multiple mechanisms may account for the protective effect of SC on axonal damage, and therefore the different studies do not seem conflicting but rather complementary. Yet, it is interesting that this manuscript shows that the myelination status of SCs does not impact their ability to slow down degeneration and yet it confirms that different timing after injury elicits different behaviors in SCs, as suggested by the studies of Babetto et al., 2020 and Vaquie et al., 2019. In other words, a more accurate description of the results of these two studies is needed and a better explanation of what the authors consider to be conflicting and why (there could be more differences than species and myelination, for instance, such as the method used for axotomy
- laser vs cut with scalpel which tear and pull membranes).

Overall, the title does not appear to be the most appropriate because the content rather proposes a detailed protocol and gives examples of applications, rather than focusing on the protective versus destructive role of SCs on axons. It also appears to be misleading, as "axo- destructive" appears to suggest a negative role of Schwann cells on axons, whereas SC are rather helpful in clearing degenerative axons, a step which facilitates regeneration.

The number of biological replicates for each experiment is not always indicated, and if the "n="

© 2023. Published by The Company of Biologists under the terms of the Creative Commons Attribution License (https://creativecommons.org/licenses/by/4.0/).

represent cultures prepared independently/passaged or wells/cell. It is essential to be rigorous and clearly indicate the number of technical replicates and biological samples throughout the manuscript and provide a thorough description of them. One example is Fig. 4 E were only 10 cells from a single culture appeared to have been imaged. Is this accurate? This aspect is essential to evaluate reproducibility, especially in view of the technical and biological variability.

Minor comments:

- Does myelination reach axons in the microgrooves (it seems to from 2C, but up to where)? Where is axotomy performed and are axons myelinated where the cut was performed?
- Since the model allows for comparison of aligned vs myelinating SCs, and that both aligned and myelinating SCs seem to slow down degeneration, and that c-JUN is upregulated after in vivo injury, have the authors measured if c-JUN levels increase similarly in both myelinating vs aligned SCs?
- On clarity:
- In the step-by-step protocol, wording needs to be improved.
- Temperatures for centrifugations are missing.
- The MOI described for lentivirus is 2-10 in the protocol but 200 in the legend of Figure 3F.
- Certain citations in the references list are incomplete (i.e. Babetto et al.; Catenaccio et al.,).

Significance

The advance for the field proposed by this paper is mostly technical, as it details a new model to be used by the field, of mouse SCs-mouse DRGs in dissociating myelinating cultures. The tested applications allowed the authors to also confirm a protective role for SCs on axonal damage, which was independent from myelination status.

Being a method paper, it is essential that the authors provide clear statements on the number of biological replicates, and technical repeats, as well as a very thorough and accurate description of the methodology.

The model described has similarities with existing models in the field such as Stevens et al., 1998 and Vaquié et al., 2019. To place it in context in a more helpful way, the authors should emphasize on the novelty brought by their protocol compared to existing models. The authors compare their findings to results from Vaquié et al., 2019 and Babetto et al., 2020 that they describe as conflicting, when it seems they rather address different mechanisms of SCs in protection and repair, occurring at different time points.

Audience might be interested in the detailed step by step protocol to use this in vitro model for the applications described, and investigate further why SCs myelination status does not influence their ability to protect from neurodegeneration early on or how to make use of this for neuroprotection studies.

Author response to reviewers' comments

Author's response to reviewers

Reviewer #1 (Evidence, reproducibility and clarity (Required)):

Summary:
In this original article from Mutscher et al., the authors developed a compartmentalized dissociated mouse myelinating SC-DRG coculture system to investigate the distinct roles of Schwann cells in axon protection and degeneration after injury. The innovation of this approach relies on (i) the use of mouse SCs and mouse DRGS neurons instead of rat cells; (ii) the use of microfluidic chambers, seeded by axons and SCs in different compartment; (iii) the possibility

© 2023. Published by The Company of Biologists under the terms of the Creative Commons Attribution License (https://creativecommons.org/licenses/by/4.0/).

to perform a traumatic injury in vitro. While this novel approach offers new ways to study peripheral nerve regeneration and SC-axon interaction, and technical the study is robust, the paper is currently limited by the exploration of their model.

Major points:
Reviewer 1.
It is unclear is this approach will ever lead to the identification of key mechanism or key candidates. This is a major miss in the current manuscript form. In short: the authors should demonstrate that their in vitro system can lead to significant leap in our understanding of peripheral nerve regeneration by identifying novel targets/pathways or mechanisms.

Author response: We agree with the reviewer that cell culture approaches have limitations however we would disagree that it is not a viable approach given that a number of seminal studies in the field have already helped identified key cellular and molecular steps using rat SC-DRG cocultures or using mouse DRGs and rat SCs in combination with in vivo study. We have added the following to the introduction to highlight this point in more detail:

Introduction.
Dissociated myelinating SC-DRG cocultures from rats were first developed by the Bunge laboratory in the 1980's to investigate PNS myelination in a more dynamic way (Bunge et al, 1989; Eldridge et al, 1987). These cultures have been used to make seminal discoveries in uncovering the cellular and molecular mechanisms of SC myelination alongside in vivo investigation. These include how the inner SC membrane (mesaxon) advances to myelinate axons, and the role of ß-neuregulin-1 (ßNRG1) and polarity proteins in SC myelination (Bunge et al, 1989; Shen et al, 2014; Chan et al, 2006; Taveggia et al, 2005). Similarly, SC- DRG cocultures have been useful in demonstrating how SCs proliferate after axon injury, transfer metabolites, such as pyruvate, to delay axon degeneration, how placental growth factor (Plgf) regulates axon fragmentation by SCs and how SC JUN promotes axon outgrowth after injury (Arthur-Farraj et al, 2011; Babetto et al, 2020; Vaquié et al, 2019; Salzer & Bunge, 1980). The use of a coculture system to study axon-SC interactions during axon degeneration and regeneration offers some advantages over in vivo approaches as both neurons and SCs can be genetically manipulated separately and live imaged with ease.

Discussion.
Most importantly SCs and DRG neurons from various transgenic mice can be used to perform in vitro analysis to complement findings from in vivo transgenic mouse studies.

Author response: Furthermore, as this is a methods paper, demonstrating novel molecular mechanisms is outside the scope of this article. However, we have already used this technique with a collaborator to study the role of cdk7 in myelination (see link to conference abstract below) and this manuscript is under preparation to be submitted soon. Additionally, we have ongoing projects within the lab using this technique to help characterise novel molecular targets in nerve injury.
https://scholar.google.com/citations?view_op=view_citation&hl=en&user=uEtwAd8AAAAJ&sortby=pubdate&citation_for_view=uEtwAd8AAAAJ:_Qo2XoVZTnwC

Author response: Despite this, we have shown that the axo-protective effect of SCs is independent of myelination status, which is an advance on what is known in the literature.

Author response: We have realised through all of the reviewers' comments that the title and the aims of the manuscript were confusing. We have made this clearer by removing the word novel in the title changing the title to the following:

A method for mouse myelinating Schwann cell-DRG neuron compartmentalised cocultures: myelination status does not influence the axo-protective effect of Schwann cells after injury.

Author response: We have also made it much clearer what the purpose of our study is and

© 2023. Published by The Company of Biologists under the terms of the Creative Commons Attribution License (https://creativecommons.org/licenses/by/4.0/).

where and how it fits in with the previous literature by adding the following paragraph to the introduction.

Introduction.
Indeed there has only ever been one laboratory detailing convincing myelin formation in dissociated mouse myelinating SC-DRG neuron cocultures, however this was never published as a step by step detailed protocol (Stevens et al, 1998; Stevens & Fields, 2000). In the last twenty years, there have been no published studies demonstrating myelination in fully dissociated mouse SC-mouse DRG cocultures. This has largely prevented the use of cells, particularly SCs, from transgenic mice in cocultures and thus restricted the ability to study SC-axon interactions in a system that can be readily manipulated and live imaged and results directly applied back to in vivo findings in the same species.

Reviewer 1. The use of embryonic DRG neurons or SC isolated from P2 animals are arguably physiologically not the same cells that are affected by traumatic nerve injury, which happen most often than not in adult. This is a problem in the long-term reliance on this approach to study axotomy peripheral nerve regeneration.

Author response: We agree with the reviewer that one should always be cautious with the use of embryonic/neonatal cells to directly refer them to adult cellular mechanisms. We have added discussion of this point to the discussion:

Discussion.
One limitation of our coculture model and indeed all coculture and cell culture models that are used to investigate cellular and molecular mechanisms in nerve injury is that the cells are obtained from embryonic or neonatal animals. This is an important caveat when applying results from cell culture to adult in vivo nerve injury. However, while we would argue that cell culture approaches should always be used in combination with in vivo study it is important to remember that nerve injury is not restricted to adults and brachial plexus injury secondary to birth trauma is unfortunately a significant clinical problem (Pondaag et al, 2007). Furthermore, neonatal SCs replicate many of key cellular and molecular mechanisms seen in adult SCs after injury, including JUN upregulation, myelinophagy, promotion of axon growth and expression of key repair program transcripts (Arthur-Farraj et al, 2012; Gomez- Sanchez et al, 2015; Arthur-Farraj et al, 2017; Parkinson et al, 2008). A future development would be to try to adapt this protocol to make a coculture model with adult mouse or even human cells.

Author response: Additionally, we already know Schwann cells in P5 neonatal mice in vivo after nerve transection demyelinate in a similar way to Schwann cells in adult mice and that neonatal cells *in vivo* and *in vitro* require the transcription factor c-Jun to do so (Parkinson et al., 2008 JCB Fig.7).

horse serum and presumably plated their cultures on laminin, though they do not explicitly detail this (Stevens et al, 1998). In our preliminary experiments we were unable to visualise much myelination with use of laminin, ascorbic acid or indeed if ßNRG1 and high concentration forskolin was added to the medium for up to four weeks. However, if we plated cocultures on Matrigel® and continuously added it to the myelination medium then we saw comparable levels of myelination in our mouse cocultures to that of rat cocultures (Eldridge et al, 1987). This approach of using Matrigel® to enhance myelination has previously been successfully employed in cultures of human iPSC sensory neurons with rat SCs and in non- dissociated mouse DRG explant cultures (Clark et al, 2017; Päiväläinen et al, 2008). Importantly, we used growth factor depleted Matrigel® as standard Matrigel® preparations contain substantial amounts of Transforming growth factor ß (TGFß) which is a known inhibitor of myelination (Einheber et al, 1995). Additionally, the majority of rat and mouse coculture protocols plate cells on glass whereas we found cultures were healthier and myelinated better when cultured on plastic Alcar® coverslips.

Discussion
Furthermore, as this is a dissociated and compartmentalised purely mouse cell culture system, one can utilise the vast array of transgenic and knockout lines available to study neuron-SC interactions in more detail, without concern of contaminating endogenous SCs and

© 2023. Published by The Company of Biologists under the terms of the Creative Commons Attribution License (https://creativecommons.org/licenses/by/4.0/).

other non-neuronal cells that remains a drawback of current mouse dissociated or non-dissociated DRG explant models.

Reviewer 1: The figures in the paper are largely descriptive. They are very little quantitative measurement. Thus, the readers will have a hard to determine, if they replicate the proposed approach, whether their efficient is on par with the current authors.

Author response: We have now added quantification of myelin segments per mm^2, percentage of SCs that myelinate and quantification of the interperiodic distance of the myelin formed. This is all included in a new version of TABLE 1. We have discussed this data in the results section as follows.

Results

Quantifying the number of PRX positive myelin segments we found that there were 325.33 ± 12.3 sheaths per mm^2, comparable to what has been originally described in rat SC/DRG cocultures and two fold more extensive myelination than recently described compartmentalised rat cocultures models (n=3; Table 1; Eldridge et al, 1987; Vaquié et al, 2019). Furthermore, 25.47 ± 1% of Schwann cells were myelinating in our cultures (n=3; Table 1). To confirm that cocultured myelinated SCs formed compact myelin we performed electron microscopy (EM), which revealed compact myelin formation with multiple myelin wraps and formation of readily visible major dense (MDL) and intraperiod lines (IPL; Fig. 2D). Additionally, we measured the periodicity, i.e., the distance between two adjacent major dense lines, to make sure myelin was compacted. Interperiodic distance was 12.16 ± 0.28 nm, in line with previous reports (n=3; Table 1; Boutary et al, 2021; Fernando et al, 2016; García-Mateo et al, 2018; Giese et al, 1992; Perrot et al, 2007).

Author response: Interestingly, after we performed this analysis, we realised we have double the level of myelination in our mouse cultures (325.33 ± 12.3 per mm^2) than in the compartmentalised myelinating rat cocultures in Vaquie et al., 2019 (147 ± 27 internodes per mm^2 (n = 3)).

Author response: We have also quantified the JUN upregulation after injury in both myelinating and aligned cocultures. See Fig. 3B-E).

Results

Additionally, we noted a strong upregulation of JUN protein in SCs 12 hours after axotomy (Fig. 3B and C). We also saw significant JUN upregulation 12 hours after axotomy in cocultures with aligned SCs (Fig. 3D and E).

Author response: The above quantification is in addition to the quantification of the rate of axon degeneration in the presence and absence of aligned and myelinating Schwann cells in Fig. 4B. We have also quantified the % of Schwann cells that contained axonal debris after injury – this data is now quoted in the text as we removed Fig. 4E.

We thank the reviewer for asking for additional quantification as this has improved the manuscript.

Minor points:
Fig.4B and E should show individual data points.

Author response: We have added the individual data points to Fig.4B. We have removed Fig.4E and instead quoted the data in the results section as follows:

Results

When we quantified this phenomenon, we found that 97.84 ± 1.462% (n=2) of SCs in our cocultures contained mCherry-labelled axonal fragments.

© 2023. Published by The Company of Biologists under the terms of the Creative Commons Attribution License (https://creativecommons.org/licenses/by/4.0/).

Reviewer #1 (Significance (Required)):

In addition, to the demonstration of feasibility of this in vitro approach, the main finding by the authors is that SCs have a role in neuronal protection and support is key for peripheral nerve regeneration. Thus while in vitro approach does not add key information that do not already exists in the field, it somewhat confirms that the effect is SC autonomous. Overall the approach is interesting and has potential, but the study currently lack a demonstration of its usefulness to the community.

It would have been interesting to have the authors discuss the advantages of their approach in comparison to other innovative approaches to study SC-axon interactions that have been developed in the last decade (i.e., 3D environment, microfluidic approach, transwell systems). There is also a lack of citations about similar studies in the field.

Author response: We direct the reviewer to the following paragraphs in the introduction and the discussion, which we have now elaborated on further post peer review. We discuss all relevant cocultures studies in mouse as well as all the relevant microfluidic studies and 3D coculture studies as well as the one human nerve organoid study. We found two additional studies, Numata-Uematasu 2023 using DRG mouse explant cultures and Park et al., 2021 using motorneuron-SC cocultures which we have now added to the discussion. We also briefly discuss transwell studies to assess migration as the reviewer requested.

We also discuss in detail the two microfluidic coculture injury studies Babetto et al., 2020 and Vaqiue et al., 2019 extensively throughout the manuscript. We have added further discussion of the similarities and differences between theirs and our approach in the discussion.

Introduction.

Protocols exist where endogenous mouse SCs are used to myelinate dissociated or non-dissociated DRG explant cultures. (Shen et al, 2014; Harty et al, 2019; Sundaram et al, 2021; Stettner et al, 2013; Numata-Uematasu et al, 2023). Furthermore, another protocol seeded exogenous SCs onto non-dissociated DRG explant cultures (Päiväläinen et al, 2008). Other laboratories seed cultured rat SCs onto dissociated mouse DRG axons (Taveggia & Bolino, 2018). Use of dissociated or non-dissociated DRG explants cultures preclude many experimental uses, such as using SCs from different transgenic animals and separate transfection of SCs and neurons with viruses for live imaging or genetic manipulation, and easy use of microfluidic chambers to allow injury studies and separate drug treatments to neurons or SCs. The reason for this is that antimitotics cannot be used in dissociated or non-dissociated DRG explant cultures as this depletes SCs, and the culture quickly becomes contaminated with other non-neuronal cell types, such as satellite cells and fibroblasts migrating out of the DRG. Furthermore, use of exogenous SCs in a non- dissociated DRG explant culture risks, after a period of antimitotic exposure, which was developed by Päiväläinen et al., 2008 still risks potential contamination from endogenous SCs and satellite glia migrating out of the DRG explant over time. This occurs because antimitotic treatment is unlikely to fully penetrate the whole DRG without prior dissociation. Additionally, a compartmentalised culture system cannot be readily used with non-dissociated DRG explant cultures (Päiväläinen et al, 2008).

Discussion.

Furthermore, our protocol is complementary to the recently described 3D mouse myelinating SC-motor neuron coculture system using collagen hydrogels (Hyung et al, 2021; Park et al, 2021). It will be interesting in the future to up titrate the concentration of Matrigel®, which is similar to collagen hydrogels, in our cultures to see whether further increasing extracellular matrix viscosity and stiffness improves our myelination efficiency even further. While it is possible to study cell migration in microfluidic cell culture devices, transwell models offer significant advantages to study this cellular phenomenon (Negro et al, 2022). To date, there have been no published studies of successful myelination in human SC-neuron coculture systems. Despite this rat SCs have been shown to readily myelinate human-induced pluripotent stem cell (iPSC)-derived sensory neurons and an iPSC-derived peripheral nerve organoid system which does contain myelinating SCs has recently been described (Clark et al, 2017; Van Lent et al,

© 2023. Published by The Company of Biologists under the terms of the Creative Commons Attribution License (https://creativecommons.org/licenses/by/4.0/).

2022).

These findings confirm the observations of both Babetto et al., 2020 and Vaquié et al., 2019 who used rat SCs in similar microfluidic culture systems (Vaquié et al, 2019; Babetto et al, 2020). We have shown that the axo-protective observation seen by Babetto et al., 2020 does not rely on myelination status, which was an outstanding question from that study (Babetto et al, 2020). Furthermore, in an advance from previous studies, we have visualised the axo-protective and axon clearance phenomena in the same culture and shown that they are temporally separated, with axon fragmentation and debris clearance by SCs occurring at much later timepoints after axotomy. Several different culture and experimental conditions preclude direct comparison of our study with both those of Vaquié et al., 2019 and Babetto et al., 2020. These include the use of rat SCs in both prior studies and that Babetto et al., 2020 mixed rat SC with mouse DRG axons; length of time in culture, time points quantified after injury and distance from injury and site of analysis. Babetto et al., 2020 performed axotomy on relatively short term cocultures (6 days in vitro) whereas Vaiquié et al., 2019 cultured for at least 4 weeks and in our case 6 weeks prior to axotomy. Vaiquié et al., 2019 removed nerve growth factor (NGF) prior to laser axotomy and quantified proximally (though also imaged distally) whereas both our study and Babetto et al., 2020 kept NGF in the medium, performed axotomy with a scalpel and quantified more distally and in our case extremely distal, where only individual neurites and no axon bundles were visible. Vaquié et al., 2019 had SCs on both sides of the barrier in the microfluidic chambers whereas we seeded SCs only in the axonal/bottom compartment. Additionally, our cocultures had both forskolin and ßNRG1 added to help induce myelination, whereas these factors are not required in rat myelinating cocultures. Finally, it is important to permeabilise myelinated cultures with acetone aŌer fixaΘon, as we did, to visualise the entire axon through heavily myelinated segments otherwise axon integrity cannot be reliably assessed in a quantitative manner (Vaquié et al, 2019; Babetto et al, 2020).

Because of the lack of key novel mechanisms, and lack of discussion on what this approach is superior to others in vitro approach limits the impact of the study and the excitement of the reader, even from the SC-axon community.

Author response: We have developed the first compartmentalised fully dissociated mouse myelinating coculture system in over twenty years. Thanks to the reviewer's suggestions, we have now shown that myelination is comparable to the original rat cocultures from the Bunge lab, which is the gold standard in the field, and superior to recently described compartmentalised rat coculture system by Vaquie et al., 2019. We have provided a detailed step by step protocol to allow other researchers to use our technique.

Additionally, thanks to the reviewer, we have now described in detail exactly how our protocol differs from others and why we succeeded to get mouse SCs to myelinate so robustly in a fully dissociated coculture (see previous answers). This is an incremental but important advance given that studies currently use a coculture system using entirely cells from rat, or where rat Schwann cells are seeded on mouse axons, or dissociated or non- dissociated mouse explant cultures are used which abrogates using neurons and SCs from different transgenic mice.

As this is a methods paper, we did not intend to describe novel molecular mechanisms though our method is already being used for such purposes by ourselves and a collaborator as outlined above in prior answers. We did not make this clear and we hope the extensive revision of the manuscript now addresses this point. Despite this, we have shown that the axo-protective effect of SCs is independent of myelination status, which is an advance on what is known in the literature.

Finally, in addition to myelination, we have demonstrated that one can study all the key components of the SC and axonal response to injury in a quantifiable way in addition to demonstrating that these cocultures can be live imaged and used for drug studies. None of the prior mouse studies looked at injury responses of axons nor SCs. We believe this will be of use to the community.

© 2023. Published by The Company of Biologists under the terms of the Creative Commons Attribution License (https://creativecommons.org/licenses/by/4.0/).

Reviewer #2 (Evidence, reproducibility and clarity (Required)):

In the manuscript entitled "Distinct axo-protective and axo-destructive roles for Schwann cells after injury in a novel compartmentalised mouse myelinating coculture system", Arthur- Farraj and colleagues detail a method of dissociated coculture of mouse-DRG neurons and Schwann cells in microfluidic chambers. In this system, neurons and Schwann cells harvested from the same or from different animals are grown in different compartments that are connected by microgrooves, thereby allowing for spatial and diffusive separation. Neurons are shown to extent their axons across the microgroove barrier to the glial compartment where Schwann cells align with the axons and myelination can be induced. Detailed analysis of myelination and axon injury/degradation are presented as use cases, including the capability to genetically and pharmacologically manipulate neurons and Schwann cells independently, which also enabled fluorescent life cell imaging. The authors then examine the effect of immature/premyelinating and myelinating Schwann cells on the rate of axon degeneration. Upon axotomy Schwann cells significantly delayed degeneration, with no difference between non-myelinating and myelinating Schwann cells. Finally, live imaging during axon degeneration with fluorescent proteins separately expressed in neurons and Schwann cells demonstrated that Schwann cells ingest axonal fragments.

Major comment:
In establishing a much needed in-vitro system for PNS myelination and injury research the paper represents a valuable contribution to the PNS community. However, I find the presentation of aspects concerning a protective/destructive role of Schwann cells somewhat inconclusive. That these roles exist has been known, as the authors discuss. Then what does this study contribute concerning the open question that was raised by the discrepancies between Babetto et al., 2020 and Vaquié et al., 2019, i.e. how Schwann cells contribute to axon survival/regeneration after injury? Essentially, the only significant conclusion in this regard is that myelinating and non-myelinating mouse Schwann cells do not differ in their capability to protect axons from degeneration. The manuscript, including the title, would benefit from focusing more on this aspect. In particular, the discussion of the factors that lead to the still remaining apparent discrepancies between Babetto et al., 2020 and Vaquié et al., 2019 and this study should be revised. The authors state that "The study by Vaiquié et al., 2019 quantified axon fragmentation proximally in the microgrooves at timepoints starting from 12 hours after axotomy." (Discussion). While this observation is accurate, Jacob and colleagues also show accelerated, obviously distal axon degeneration in the presence of Schwann cells (Figure 3C in Vaquié et al., 2019). It is therefore unlikely that the discrepancies stem from analysis of more proximal vs. more distal axons, or the timepoints of analyses. In my opinion, a further study (using the coculture system presented in this manuscript) that compares the role of Schwann cells from rats and mice, and that includes analysis of more proximal and distal axon degeneration as well analysis of axon regeneration is needed. In a rework of the manuscript, the authors may therefore elaborate more on the shortcomings of the present study, or alternatively soften the aims of the study in the first place.

Author response: We thank the reviewer for their comments, and we agree that the title and the aims of the manuscript were confusing. We didn't make it explicit that this was a methods paper, and that we didn't intend to show entirely novel findings but instead thoroughly characterise our mouse system so that it is comparable to what has been done for rat cocultures. We have now made this clearer by removing the word novel in the title and changing the title to the following:

A method for mouse myelinating Schwann cell-DRG neuron compartmentalised cocultures: myelination status does not influence the axo-protective effect of Schwann cells after injury.

Author response: We have decided to focus the manuscript more on the comparison of myelinating versus non-myelinating cocultures, given that we have shown that the axo-protective effect of SCs is independent of myelination status, which is an advance on what is known in the literature. We have added further characterisation of our aligned cocultures with p75NTR immuno and EM images (Fig.1D and E). We have added the following summary of how our study relates to findings of Babetto et al., 2020 and Vaquie et al., 2019 in the discussion, in

© 2023. Published by The Company of Biologists under the terms of the Creative Commons Attribution License (https://creativecommons.org/licenses/by/4.0/).

line with the reviewer's suggestions.

Discussion.
These findings confirm the observations of both Babetto et al., 2020 and Vaquié et al., 2019 who used rat SCs in similar microfluidic culture systems (Vaquié et al, 2019; Babetto et al, 2020). We have shown that the axo-protective observation seen by Babetto et al., 2020 does not rely on myelination status, which was an outstanding question from that study (Babetto et al, 2020). Furthermore, in an advance from previous studies, we have visualised the axo-protective and axon clearance phenomena in the same culture and shown that they are temporally separated, with axon fragmentation and debris clearance by SCs occurring at much later timepoints after axotomy.

Author response: We have now added more in-depth discussion of the similarities and differences between Babetto et al., 2020 and Vaquie et al., 2019 and our approach in the discussion.

Discussion.
Several different culture and experimental conditions preclude direct comparison of our study with both those of Vaquié et al., 2019 and Babetto et al., 2020. These include the use of rat SCs in both prior studies and that Babetto et al., 2020 mixed rat SC with mouse DRG axons; length of time in culture, time points quantified after injury and distance from injury and site of analysis. Babetto et al., 2020 performed axotomy on relatively short term cocultures (6 days in vitro) whereas Vaiquié et al., 2019 cultured for at least 4 weeks and in our case 6 weeks prior to axotomy. Vaiquié et al., 2019 removed nerve growth factor (NGF) prior to laser axotomy and quantified proximally (though also imaged distally) whereas both our study and Babetto et al., 2020 kept NGF in the medium, performed axotomy with a scalpel and quantified more distally and in our case extremely distal, where only individual neurites and no axon bundles were visible. Vaquié et al., 2019 had SCs on both sides of the barrier in the microfluidic chambers whereas we seeded SCs only in the axonal/bottom compartment. Additionally, our cocultures had both forskolin and ßNRG1 added to help induce myelination, whereas these factors are not required in rat myelinating cocultures. Finally, it is important to permeabilise myelinated cultures with acetone aŌer fixaΘon, as we did, to visualise the entire axon through heavily myelinated segments otherwise axon integrity cannot be reliably assessed in a quantitative manner (Vaquié et al, 2019; Babetto et al, 2020).

Author response: We would like to add that we showed Claire Jacob, senior author of the Vaquie et al., 2019 study, our manuscript prior to peer review and she offered helpful comments, which we incorporated into the manuscript, which is why she is acknowledged. We have also discussed our findings with Elisabetta Babetto as well.

While it would be a great future study to compare both axon degeneration rates in rat and mouse cocultures this was not the original intention of our study. We believe we have included enough detail of our experimental procedures, including the distance from the barrier we imaged axon degeneration, a crucial bit of information missing from the other studies, should others want to perform a comparative study between rats and mice.

Methods
Five images at a distance of between 1.2-1.4mm from the microgroove barrier (the most distal part of the culture that could be imaged) were quantified per culture, taken in comparable locations in each culture.

Author response: I would add that one of our previous studies (Arthur-Farraj et al., 2012 Neuron, Fig5I) has already looked at axon outgrowth/regeneration in dissociated non-myelinating mouse SC-DRG co-cultures. We showed the presence of Schwann cells accelerates axon regeneration/outgrowth and this relies upon Schwann cell c-JUN.

We have now added quantification of the extent of myelination in our cocultures and it is comparable to the original Bunge lab rat cocultures and more extensive than the Vaquie et al., 2019 coculture.

© 2023. Published by The Company of Biologists under the terms of the Creative Commons Attribution License (https://creativecommons.org/licenses/by/4.0/).

Results
Quantifying the number of PRX positive myelin segments we found that there were 325.33 ± 12.3 sheaths per mm^2, comparable to what has been originally described in rat SC/DRG cocultures and two fold more extensive myelination than recently described compartmentalised rat cocultures models (n=3; Table 1; Eldridge et al, 1987; Vaquié et al, 2019). Furthermore, 25.47 ± 1% of Schwann cells were myelinating in our cultures (n=3; Table 1).

Methods
To quantify the number of myelin segments per area, we counted the number of myelin segments for five areas per culture for three cultures and normalised this per mm^2. To quantify the percentage of Schwann cells in myelinating cocultures that are actively myelinating, we quantified the number of myelin segments and the number of DAPI-positive nuclei for five areas per culture for three cultures.

Minor comments:
- Figure 2D: From the electron micrograph there is no doubt that compact myelin is formed, however to me it seems the compaction is not complete. A rough estimation with the aid of the provided scale bar resulted in an interperiodic distance of about 17 nm, which contrasts with the remarkably well reproduced values reported in multiple reports using conventional specimen preparation (like in this study), of which I am citing just a few: about 13 nm in rat ex vivo nerve (Peterson and Pease, J. Ultrastruct Res 1972; Fledrich et al., Nat Commun 2018), 12 nm (Giese et al., Cell 1992), 12.2 nm (Perot et al., J Neurosci 2007), about 12 (Fernando et al., Nat Commun 2016), about 13 nm (García-Mateo et al., Glia 2017) or about 13 nm in mouse ex vivo (Boutary et al., Commun Biol 2021), which was also reproduced with about 13 nm in rat in vitro (Taveggia and Bolino, Methods Mol Biol 2018). The authors should acknowledge this deviation and might discuss possible reasons.

Author response: We have now provided a more representative EM image of our myelination (Fig. 2D). Additionally, thanks to the reviewer's comments we have now quantified the interperiodic distance and find it is comparable to the studies the reviewer suggested. We have added the data to the new Table 1 and added the references the reviewer advised. Please see the additions to the methods and the results section regarding this new data below.

Results
To confirm that cocultured myelinated SCs formed compact myelin we performed electron microscopy (EM), which revealed compact myelin formation with multiple myelin wraps and formation of readily visible major dense (MDL) and intraperiod lines (IPL; Fig. 2D). Additionally, we measured the periodicity, i.e., the distance between two adjacent major dense lines, to make sure myelin was compacted. Interperiodic distance was 12.16 ± 0.28 nm, in line with previous reports (n=3; Table 1; Boutary et al, 2021; Fernando et al, 2016; García-Mateo et al, 2018; Giese et al, 1992; Perrot et al, 2007).

Methods
To measure interperiodic distance, we measured at least 10 periods per myelinated fibre for at least three fibres per sample for three separate samples.

- Figure 4A,B: The result of a slowed axon degeneration in coculture relies on the accurate assessment of continuity of the NFL staining. While the authors report that acetone permeabilization was necessary to afford complete penetration of the used antibodies in myelinating cultures, I cannot see why the authors have not used the same staining protocol for all cultures, as it is detailed in the method section. While I consider it unlikely that the staining conditions have led to an apparent delay of degeneration in coculture, experiments should generally be performed under identical conditions, unless there are good reasons not to do so. If this is not the case, it will be reassuring to see the same effect when identical staining conditions are employed. On the same note, do the compared cultures have the same age, i.e. have the neuron monocultures been in vitro for the same time as the cocultures?

Author response: We thank the reviewer for picking up this inaccuracy in the manuscript. We can confirm that for the purposes of the axon degeneration experiment all cultures were

© 2023. Published by The Company of Biologists under the terms of the Creative Commons Attribution License (https://creativecommons.org/licenses/by/4.0/).

stained using exactly the same staining protocol. Additionally, we were very careful to maintain all cultures for exactly the same time in culture – 6 weeks. Additionally, axon only cultures were maintained in myelination medium to make sure medium constituents were not responsible for the observed differences in degeneration rate. We have added the following elaboration to the methods section to clarify these points.

Methods
Axon only cultures related to Figure 1 were permeabilised in PBS + 0.5% Triton (Merck) + 5% HS + 5% donkey serum (DS, Merck - D9663) at RT for 1 hour. For the purposes of quantifying the rate of axon degeneration (Figure 4) both axon only cultures and cocultures with SCs were permeabilised in 50% Acetone for 2 minutes, 100% Acetone for 2 minutes, 50% Acetone for 2 minutes (all at RT), and then blocked in PBS + 0.5% Triton + 5% HS + 5% DS at RT for 1 hour.

Methods
All cultures (axon only, aligned SCs and myelinating SCs) were cultured for 6 weeks prior to axotomy experiments. To minimise the possibility that medium constituents were responsible for differences in axon degeneration rates, axonal compartments of axon only cultures were cultured in medium containing 10 ng ml^{-1} ßNRG1 and 10 µM forskolin (axon only medium, extended methods section D6) once SCs were seeded on other cultures, and then switched into myelination medium (additional Matrigel and 50 µg ml^{-1} L-Ascorbic Acid), 24 hours before axotomy. Bottom compartments of aligned SC cultures, 24 hours before axotomy, were switched into DRG/SC medium containing 10 ng ml^{-1} ßNRG-1, 10 µM forskolin and 50 µg ml^{-1} L-Ascorbic Acid, which is insufficient to induce myelination in mouse cultures. Bottom compartments of myelinating cocultures were medium changed into fresh myelination medium (Extended methods section D7) 24 hours prior to axotomy.

- In several instances of the manuscript, the term "transfection" is used to refer to lentiviral gene transfer. I advise to use the more appropriate term "transduction" instead
- I could not seem to find a meaningful reference to the microfluidic chambers that were used in the study. The protocol should contain details on the device and source of supply in order to enable potential readers to execute the protocol

Author response: We thank the reviewer for this comment. We have replaced transfection with transduction throughout the manuscript. Please see the track changes manuscript for all instances.

Reviewer #2 (Significance (Required)):

The paper presents a convincing establishment of a dissociated coculture derived exclusively from mouse that leads to robust myelination. As the manuscript correctly states, Schwann cell culture and especially coculture with neurons has been experienced difficult in the field, and by providing a detailed protocol as well as demonstrating how the coculture system can be used to address important questions of PNS myelination and repair, the paper fills an important gap. However, the experiments directed to the role of Schwann cells in axon degeneration do not clarify much, which should be better addressed in the discussion and also by modifying the title accordingly.
The paper will be of high value for basic researchers that are interested in performing studies addressing cellular and molecular mechanisms of myelination and repair in the PNS. Importantly, the paper can pave the way to usage of transgenic or knockout mouse models in coculture. Thereby it might spark interest also in those researchers that use transgenic and knockout mouse models and who have so far refrained from using coculture models.

Field of expertise of the reviewer: Cellular and molecular mechanisms of myelination and growth signaling in the PNS; in-depth experience with DRG coculture models from rats and mice

Author response: We thank the reviewer for their kind comments. We have now modified the title, aims and discussion of the manuscript in line with the reviewer's suggestions.

© 2023. Published by The Company of Biologists under the terms of the Creative Commons Attribution License (https://creativecommons.org/licenses/by/4.0/).

Reviewer #3 (Evidence, reproducibility and clarity (Required)):

SUMMARY
The authors present a detailed protocol for co-cultures of mouse DRGs with mouse SCs using microfluidics. In this model, cells of interest grow in different compartments while allowing for axons to grow in between, thereby making them accessible to injury induction. Using this experimental system, the authors show that myelination occurs, myelin gets compacted and acquires nodal organization. The authors then show that such a system allows for compartment-specific lentivirus transduction and live imaging. Next, they perform physical and chemical axonal injury and show that at early time point pos- injury the presence of SCs protects from axonal degeneration regardless of the myelination status, and helps with clearing of damaged axons at later time points.

Major comments:

The novelty of the study is questionable.
While the model is well described and appears to be useful for the proposed applications (live imaging, transduction, injury model), the arguments provided regarding its novelty are not fully convincing. The main argument from the authors of this paper is that there are no established protocols describing the use of mouse SCs with mouse DRG neurons in dissociated myelinating cocultures. However, this appears to be inaccurate, as the model described in Stevens et al., 1998 (cited in the paper) uses mouse DRG neurons dissected at E13.5 with mouse SCs dissected at P3 to study myelination. Also, in Päiväläinen et al., 2008, mouse DRGs and SCs are cultured from transgenic mice at different developmental ages, thereby arguing that coculture models have been previously successfully implemented. The main difference appears to be rather the compartmentalization of SCs and DRGs which appears to be a mouse adaptation of the rat model described by Vaquie et al,2019. Based on the above, it seems imperative for the authors to tone down the novelty aspect and provide a more thorough discussion on how the current novel differs from protocols in published study, highlighting advantages and caveats for each.

Author response: We agree with the reviewer that we did not make the case clear enough for how our coculture model adds to what is currently described in the literature. We have now changed the title and removed the word novel. New title:

A method for mouse myelinating Schwann cell-DRG neuron compartmentalised cocultures: myelination status does not influence the axo-protective effect of Schwann cells after injury.

Author response: We have now added the following paragraph to the introduction:

Introduction.
Indeed there has only ever been one laboratory detailing convincing myelin formation in dissociated mouse myelinating SC-DRG neuron cocultures, however this was never published as a step by step detailed protocol (Stevens et al, 1998; Stevens & Fields, 2000). In the last twenty years, there have been no published studies demonstrating myelination in fully dissociated mouse SC-mouse DRG cocultures. This has largely prevented the use of cells, particularly SCs, from transgenic mice in cocultures and thus restricted the ability to study SC-axon interactions in a system that can be readily manipulated and live imaged and results directly applied back to in vivo findings in the same species.

Author response: Regarding the study by Päiväläinen et al, 2008, they did not fully dissociate their DRGs (see Fig.1 which demonstrates a DRG explant) and thus it is a non- dissociated DRG explant model. While they demonstrated convincing myelination due to the use of Matrigel which we acknowledge them for, their model is not perfectly suited for the use of neurons and SCs from different transgenic animals as the use of a DRG explant, even with temporary use of an antimitotic, risks contamination by endogenous SCs and satellite glia over time, especially as their model is not compartmentalised. We discuss the caveats of their protocol and those using dissociated mouse explant cocultures in a revised paragraph in the

© 2023. Published by The Company of Biologists under the terms of the Creative Commons Attribution License (https://creativecommons.org/licenses/by/4.0/).

introduction.

Introduction.

Protocols exist where endogenous mouse SCs are used to myelinate dissociated or non-dissociated DRG explant cultures. (Shen et al, 2014; Harty et al, 2019; Sundaram et al, 2021; Stettner et al, 2013; Numata-Uematasu et al, 2023). Furthermore, another protocol seeded exogenous SCs onto non-dissociated DRG explant cultures (Päiväläinen et al, 2008). Other laboratories seed cultured rat SCs onto dissociated mouse DRG axons (Taveggia & Bolino, 2018). Use of dissociated or non-dissociated DRG explants cultures precludes many experimental uses, such as using SCs from different transgenic animals and separate transfection of SCs and neurons with viruses for live imaging or genetic manipulation, and easy use of microfluidic chambers to allow injury studies and separate drug treatments to neurons or SCs. The reason for this is that antimitotics cannot be used in dissociated or non-dissociated DRG explant cultures as this depletes SCs, and the culture quickly becomes contaminated with other non-neuronal cell types, such as satellite cells and fibroblasts migrating out of the DRG. Furthermore, use of exogenous SCs in a non- dissociated DRG explant culture risks, after a period of antimitotic exposure, which was developed by Päiväläinen et al., 2008 still risks potential contamination from endogenous SCs and satellite glia migrating out of the DRG explant over time. This occurs because antimitotic treatment is unlikely to fully penetrate the whole DRG without prior dissociation. Additionally, a compartmentalised culture system cannot be readily used with non-dissociated DRG explant cultures (Päiväläinen et al, 2008).

Author response: We have also added further discussion on how our protocol differs from the Stevens 1998 and other protocols in the discussion.

Discussion

Our protocol differs somewhat from the one used by Stevens et al., 1998 to induce myelination in dissociated mouse SC-DRG cocultures, as they used ascorbic acid and 10% horse serum and presumably plated their cultures on laminin, though they do not explicitly detail this (Stevens et al, 1998). In our preliminary experiments we were unable to visualise much myelination with use of laminin, ascorbic acid or indeed if ßNRG1 and high concentration forskolin was added to the medium for up to four weeks. However, if we plated cocultures on Matrigel® and continuously added it to the myelination medium then we saw comparable levels of myelination in our mouse cocultures to that of rat cocultures (Eldridge et al, 1987). This approach of using Matrigel® to enhance myelination has previously been successfully employed in cultures of human iPSC sensory neurons with rat SCs and in in non-dissociated mouse DRG explant cultures (Clark et al, 2017; Päiväläinen et al, 2008). Importantly, we used growth factor depleted Matrigel® as standard Matrigel® preparations contain substantial amounts of Transforming growth factor ß (TGF ß) which is a known inhibitor of myelination (Einheber et al, 1995). Additionally, the majority of rat and mouse coculture protocols plate cells on glass whereas we found cultures were healthier and myelinated better when cultured on plastic Alcar® coverslips.

Author response: We have added further discussion of comparable models in the literature in the discussion.

Discussion.

Furthermore, our protocol is complementary to the recently described 3D mouse myelinating SC-motor neuron coculture system using collagen hydrogels (Hyung et al, 2021; Park et al, 2021). It will be interesting in the future to up titrate the concentration of Matrigel®, which is similar to collagen hydrogels, in our cultures to see whether further increasing extracellular matrix viscosity and stiffness improves our myelination efficiency even further. While it is possible to study cell migration in microfluidic cell culture devices, transwell models offer significant advantages to study this cellular phenomenon (Negro et al, 2022). To date, there have been no published studies of successful myelination in human SC-neuron coculture systems. Despite this rat SCs have been shown to readily myelinate human-induced pluripotent stem cell (iPSC)-derived sensory neurons and an iPSC-derived peripheral nerve organoid system which does contain myelinating SCs has recently been described (Clark et al, 2017; Van Lent et al,

© 2023. Published by The Company of Biologists under the terms of the Creative Commons Attribution License (https://creativecommons.org/licenses/by/4.0/).

2022).

Next, the authors emphasize the conflicting results of two articles, Babetto et al., 2020 and Vaquie et al., 2019, as the basis to use their newly developed model in the same species and testing two ages corresponding to distinct myelination states. However, both studies reach the same conclusion as the current study, that SCs have a protective role, although at two different developmental time points. As such, it is likely that multiple mechanisms may account for the protective effect of SC on axonal damage, and therefore the different studies do not seem conflicting but rather complementary. Yet, it is interesting that this manuscript shows that the myelination status of SCs does not impact their ability to slow down degeneration and yet it confirms that different timing after injury elicits different behaviors in SCs, as suggested by the studies of Babetto et al., 2020 and Vaquie et al., 2019. In other words, a more accurate description of the results of these two studies is needed and a better explanation of what the authors consider to be conflicting and why (there could be more differences than species and myelination, for instance, such as the method used for axotomy - laser vs cut with scalpel which tear and pull membranes).

Author response: We would like to humbly correct the reviewer that the studies by Babetto et al., 2020 and Vaquie et al., 2019 do not reach the same conclusion that Schwann cells have a protective role. Instead, they describe axon protection (Babetto et al., 2020) and axon fragmentation (Vaquie et al., 2019). Our studies now visualise both phenomena in the same culture system. We have now made this point more explicit as well as highlighted the one conceptual advance our methods paper makes on the current literature, which is that myelination status does not influence the SC axo-protection, as the reviewer suggested.

Discussion.
These findings confirm the observations of both Babetto et al., 2020 and Vaquié et al., 2019 who used rat SCs in similar microfluidic culture systems (Vaquié et al, 2019; Babetto et al, 2020). We have shown that the axo-protective observation seen by Babetto et al., 2020 does not rely on myelination status, which was an outstanding question from that study (Babetto et al, 2020). Furthermore, in an advance from previous studies, we have visualised the axo-protective and axon clearance phenomena in the same culture and shown that they are temporally separated, with axon fragmentation and debris clearance by SCs occurring at much later timepoints after axotomy.

Author response: We have now added more in-depth discussion of the similarities and differences between Babetto et al., 2020 and Vaquie et al., 2019 and our approach in the discussion.

Discussion.
Several different culture and experimental conditions preclude direct comparison of our study with both those of Vaquié et al., 2019 and Babetto et al., 2020. These include the use of rat SCs in both prior studies and that Babetto et al., 2020 mixed rat SC with mouse DRG axons; length of time in culture, time points quantified after injury and distance from injury and site of analysis. Babetto et al., 2020 performed axotomy on relatively short term cocultures (6 days in vitro) whereas Vaiquié et al., 2019 cultured for at least 4 weeks and in our case 6 weeks prior to axotomy. Vaiquié et al., 2019 removed nerve growth factor (NGF) prior to laser axotomy and quantified proximally (though also imaged distally) whereas both our study and Babetto et al., 2020 kept NGF in the medium, performed axotomy with a scalpel and quantified more distally and in our case extremely distal, where only individual neurites and no axon bundles were visible. Vaquié et al., 2019 had SCs on both sides of the barrier in the microfluidic chambers whereas we seeded SCs only in the axonal/bottom compartment. Additionally, our cocultures had both forskolin and ßNRG1 added to help induce myelination, whereas these factors are not required in rat myelinating cocultures. Finally, it is important to permeabilise myelinated cultures with acetone aŌer fixaƟon, as we did, to visualise the entire axon through heavily myelinated segments otherwise axon integrity cannot be reliably assessed in a quantitative manner (Vaquié et al, 2019; Babetto et al, 2020).

Author response: We would like to add that we showed Claire Jacob, senior author of the Vaquie et al., 2019 study, our manuscript prior to peer review and she offered helpful

© 2023. Published by The Company of Biologists under the terms of the Creative Commons Attribution License (https://creativecommons.org/licenses/by/4.0/).

comments, which we incorporated into the manuscript, which is why she is acknowledged. We have also discussed our findings with Elisabetta Babetto as well.

Overall, the title does not appear to be the most appropriate because the content rather proposes a detailed protocol and gives examples of applications, rather than focusing on the protective versus destructive role of SCs on axons. It also appears to be misleading, as "axo-destructive" appears to suggest a negative role of Schwann cells on axons, whereas SC are rather helpful in clearing degenerative axons, a step which facilitates regeneration.

Author response: We have now changed the title and the focus of the manuscript in line with the reviewer's comments. New title:

A method for mouse myelinating Schwann cell-DRG neuron compartmentalised cocultures: myelination status does not influence the axo-protective effect of Schwann cells after injury.

Author response: We have removed the phrase axo-destructive throughout the manuscript and instead referred to axon fragmentation and axon debris clearance roles of SCs in line with the reviewer's suggestion. Please see track changes manuscript for all instances where this was modified.

The number of biological replicates for each experiment is not always indicated, and if the "n=" represent cultures prepared independently/passaged or wells/cell. It is essential to be rigorous and clearly indicate the number of technical replicates and biological samples throughout the manuscript and provide a thorough description of them. One example is Fig. 4 E were only 10 cells from a single culture appeared to have been imaged. Is this accurate? This aspect is essential to evaluate reproducibility, especially in view of the technical and biological variability.

Author response: We have now added quantification of myelin segments per mm^2, percentage of SCs that myelinate and quantification of the interperiodic distance of the myelin formed. This is all included in a new version of TABLE 1. We have discussed this data in the results section as follows.

Quantifying the number of PRX positive myelin segments we found that there were 325.33 ± 12.3 sheaths per mm^2, comparable to what has been originally described in rat SC/DRG cocultures and two fold more extensive myelination than recently described compartmentalised rat cocultures models (n=3; Table 1; Eldridge et al, 1987; Vaquié et al, 2019). Furthermore, 25.47 ± 1% of Schwann cells were myelinating in our cultures (n=3; Table 1). To confirm that cocultured myelinated SCs formed compact myelin we performed electron microscopy (EM), which revealed compact myelin formation with multiple myelin wraps and formation of readily visible major dense (MDL) and intraperiod lines (IPL; Fig. 2D). Additionally, we measured the periodicity, i.e., the distance between two adjacent major dense lines, to make sure myelin was compacted. Interperiodic distance was 12.16 ± 0.28 nm, in line with previous reports (n=3; Table 1; Boutary et al, 2021; Fernando et al, 2016; García-Mateo et al, 2018; Giese et al, 1992; Perrot et al, 2007).

Author response: We have discussed the number of cultures used for each quantification in the methods section. See below.

Quantification of Myelination in cocultures
To quantify the number of myelin segments per area, we counted the number of myelin segments for five areas per culture for three cultures and normalised this per mm^2. To quantify the percentage of Schwann cells in myelinating cocultures that are actively myelinating, we quantified the number of myelin segments and the number of DAPI-positive nuclei for five areas per culture for three independently prepared cultures. To measure interperiodic distance, we measured at least 10 periods per myelinated fibre for at least three fibres per sample for three separate samples.

© 2023. Published by The Company of Biologists under the terms of the Creative Commons Attribution License (https://creativecommons.org/licenses/by/4.0/).

Quantification of Degeneration
Five images at a distance of between 1.2-1.4mm from the microgroove barrier (the most distal part of the culture that could be imaged) were quantified per culture, taken in comparable locations in each culture. A line was drawn across each image, and each axon crossing this line was either scored as degenerated or intact. Images were blinded prior to quantification. A minimum of three independently prepared cultures were assessed per timepoint for each condition.

Author response: We have removed Fig.4E and instead quoted the data in the results section as follows:

When we quantified this phenomenon, we found that 97.84 ± 1.462% (n=2) of SCs in our cocultures contained mCherry-labelled axonal fragments.

Author response: We apologise as the n number for this experiment was 2 (not 10), with cells in 10 areas quantified throughout all imaging timepoints from each independently prepared culture. We have included the following description in the methods section:

To quantify number of SCs with fragments, each cell was defined as a region of interest and checked for the presence of mCherry positive fragments at all timepoints. Two separate independently prepared cultures and cells in 10 areas per culture were analysed.

Author response: Additionally for Fig. 4B we have now included individual data points from independently prepared cultures.

N numbers are included in all figure legends and always refers to independently prepare cultures/biological replicates.

We have added to the relevant figure legends (Fig.3 and 4 and Table 1) the phrase:
n number refers to independently prepared cultures from separate litters of mice.

Minor comments:

• Does myelination reach axons in the microgrooves (it seems to from 2C, but up to where)? Where is axotomy performed and are axons myelinated where the cut was performed?

Author response: Myelination occasionally reaches the beginning of the microgrooves. We didn't visualise myelination in the DRG cell body compartment. We have added the following detail to the methods section:

Traumatic axotomies were carried out by carefully removing the microfluidic chamber (SND150 and RND150, Xona Microfluidics®) from the Aclar coverslip using sterile forceps and severing axons with a surgical blade under a light microscope. Axotomies were carried out at the level of the microgroove barrier. To confirm all axons were severed, a second higher cut was performed and axons between the cut sites removed using the surgical blade.

Author response: Given this, we cannot exclude that the odd proximal myelin segment is cut, but the vast majority of axons are not myelinated at the site of cut (lower cut).

• Since the model allows for comparison of aligned vs myelinating SCs, and that both aligned and myelinating SCs seem to slow down degeneration, and that c-JUN is upregulated after in vivo injury, have the authors measured if c-JUN levels increase similarly in both myelinating vs aligned SCs?

Author response: We thank the reviewer for this suggestion. We have now quantified the JUN upregulation after injury in both myelinating and aligned cocultures as well as adding images of JUN upregulation in aligned cocultures. See Fig. 3B-E).

© 2023. Published by The Company of Biologists under the terms of the Creative Commons Attribution License (https://creativecommons.org/licenses/by/4.0/).

Additionally, we noted a strong upregulation of JUN protein in SCs 12 hours after axotomy (Fig. 3B and C). We also saw significant JUN upregulation 12 hours after axotomy in cocultures with aligned SCs (Fig. 3D and E).

Author response: We have decided to focus the manuscript more on the comparison of myelinating versus non-myelinating cocultures, given that we have shown that the axo-protective effect of SCs is independent of myelination status, which is an advance on what is known in the literature. In addition to changing the title, as we have mentioned previously, we have added further characterisation of our aligned cocultures with p75NTR immuno and EM images (Fig.1D and E).

We have
• On clarity:
- In the step-by-step protocol, wording needs to be improved.
Author response: We have substantially edited the step-by-step protocol. Please see track changes document for all specific changes in wording.

- Temperatures for centrifugations are missing.
Author response: We have added temperatures for all centrifugation steps. Please see track changes document

- The MOI described for lentivirus is 2-10 in the protocol but 200 in the legend of Figure 3F.
Author response: The MOI for DRGs was 2-10 and SCs was 200 in Figure 3F. This is described similarly in the extended methods section. DRGs are transduced much more easily than SCs.

We have added the following sentence to the results section to emphasise this point:

Importantly dissociated mouse SCs required a much higher multiplicity of infection (MOI) than dissociated mouse DRGs (see extended methods section).

- Certain citations in the references list are incomplete (i.e. Babetto et al.; Catenaccio et al.,).
Author response: We have updated the reference list.

Reviewer #3 (Significance (Required)):
Significance
The advance for the field proposed by this paper is mostly technical, as it details a new model to be used by the field, of mouse SCs-mouse DRGs in dissociating myelinating cultures. The tested applications allowed the authors to also confirm a protective role for SCs on axonal damage, which was independent from myelination status.

Being a method paper, it is essential that the authors provide clear statements on the number of biological replicates, and technical repeats, as well as a very thorough and accurate description of the methodology.

The model described has similarities with existing models in the field such as Stevens et al., 1998 and Vaquié et al., 2019. To place it in context in a more helpful way, the authors should emphasize on the novelty brought by their protocol compared to existing models. The authors compare their findings to results from Vaquié et al., 2019 and Babetto et al., 2020 that they describe as conflicting, when it seems they rather address different mechanisms of SCs in protection and repair, occurring at different time points.

Audience might be interested in the detailed step by step protocol to use this in vitro model for the applications described, and investigate further why SCs myelination status does not influence their ability to protect from neurodegeneration early on or how to make use of this for neuroprotection studies.

Author response: We have now rephrased the description of Vaquié et al., 2019 and Babetto et al., 2020 studies in line with the reviewer's suggestions. We have now added further discussion

© 2023. Published by The Company of Biologists under the terms of the Creative Commons Attribution License (https://creativecommons.org/licenses/by/4.0/).

of our model in the context of all other models in the field as we have outlined in detail in above responses.

Original submission

First decision letter

MS ID#: JOCES/2023/261557
MS TITLE: A method for mouse myelinating Schwann cell-DRG neuron compartmentalised cocultures: myelination status does not influence the axo-protective effect of Schwann cells after injury
AUTHORS: Clara Mutschler, Shaline V Fazal, Nathalie Schumacher, Andrea Loreto, Michael P Coleman, and Peter Arthur-Farraj
ARTICLE TYPE: Research Article

We have now reached a decision on the above manuscript.

To see the reviewers' reports and a copy of this decision letter, please go to: https://submit-jcs.biologists.org and click on the 'Manuscripts with Decisions' queue in the Author Area. (Corresponding author only has access to reviews.)

I am satisfied of the changes made to the manuscript in light of the comments of the three reviewers. However, i do not feel that the new title is effective. Have you considered:

Myelination status does not influence the axo-protective effect of Schwann cells after injury in compartmentalised co-cultures of mouse myelinating Schwann cell and DRG neurons.

I believe that this title conveys more effectively the message of the paper. Furthermore I disagree with the reviewers regarding the novelty. These conditions are indeed novel; hence I would make sure that the abstrat state that this is a new optimised method for mouse myelinated DRGs.

If you do not agree with any of these criticisms or suggestions please explain clearly why this is so.

First revision

Author response to reviewers' comments

Reviewer #1 (Evidence, reproducibility and clarity (Required)):

Summary:
In this original article from Mutscher et al., the authors developed a compartmentalized dissociated mouse myelinating SC-DRG coculture system to investigate the distinct roles of Schwann cells in axon protection and degeneration after injury. The innovation of this approach relies on (i) the use of mouse SCs and mouse DRGS neurons instead of rat cells; (ii) the use of microfluidic chambers, seeded by axons and SCs in different compartment; (iii) the possibility to perform a traumatic injury in vitro. While this novel approach offers new ways to study peripheral nerve regeneration and SC-axon interaction, and technical the study is robust, the paper is currently limited by the exploration of their model.

Major points:
Reviewer 1. It is unclear is this approach will ever lead to the identification of key mechanism or key candidates. This is a major miss in the current manuscript form. In short: the authors should

© 2023. Published by The Company of Biologists under the terms of the Creative Commons Attribution License (https://creativecommons.org/licenses/by/4.0/).

demonstrate that their in vitro system can lead to significant leap in our understanding of peripheral nerve regeneration by identifying novel targets/pathways or mechanisms.

Author response: We agree with the reviewer that cell culture approaches have limitations however we would disagree that it is not a viable approach given that a number of seminal studies in the field have already helped identified key cellular and molecular steps using rat SC-DRG cocultures or using mouse DRGs and rat SCs in combination with in vivo study. We have added the following to the introduction to highlight this point in more detail:

Introduction.
Dissociated myelinating SC-DRG cocultures from rats were first developed by the Bunge laboratory in the 1980's to investigate PNS myelination in a more dynamic way (Bunge et al, 1989; Eldridge et al, 1987). These cultures have been used to make seminal discoveries in uncovering the cellular and molecular mechanisms of SC myelination alongside in vivo investigation. These include how the inner SC membrane (mesaxon) advances to myelinate axons, and the role of β-neuregulin-1 (βNRG1) and polarity proteins in SC myelination (Bunge et al, 1989; Shen et al, 2014; Chan et al, 2006; Taveggia et al, 2005). Similarly, SC-DRG cocultures have been useful in demonstrating how SCs proliferate after axon injury, transfer metabolites, such as pyruvate, to delay axon degeneration, how placental growth factor (Plgf) regulates axon fragmentation by SCs and how SC JUN promotes axon outgrowth after injury (Arthur-Farraj et al, 2011; Babetto et al, 2020; Vaquié et al, 2019; Salzer & Bunge, 1980). The use of a coculture system to study axon-SC interactions during axon degeneration and regeneration offers some advantages over in vivo approaches as both neurons and SCs can be genetically manipulated separately and live imaged with ease.

Discussion.
Most importantly SCs and DRG neurons from various transgenic mice can be used to perform in vitro analysis to complement findings from in vivo transgenic mouse studies.

Author response: Furthermore, as this is a methods paper, demonstrating novel molecular mechanisms is outside the scope of this article. However, we have already used this technique with a collaborator to study the role of cdk7 in myelination (see link to conference abstract below) and this manuscript is under preparation to be submitted soon. Additionally, we have ongoing projects within the lab using this technique to help characterise novel molecular targets in nerve injury.
https://scholar.google.com/citations?view_op=view_citation&hl=en&user=uEtwAd8AAAAJ&sortby=pubdate&citation_for_view=uEtwAd8AAAAJ:_Qo2XoVZTnwC
Author response: Despite this, we have shown that the axo-protective effect of SCs is independent of myelination status, which is an advance on what is known in the literature.

Author response: We have realised through all of the reviewers' comments that the title and the aims of the manuscript were confusing. We have made this clearer by removing the word novel in the title changing the title to the following:

A method for mouse myelinating Schwann cell-DRG neuron compartmentalised cocultures: myelination status does not influence the axo-protective effect of Schwann cells after injury.

Author response: We have also made it much clearer what the purpose of our study is and where and how it fits in with the previous literature by adding the following paragraph to the introduction.

Introduction.
Indeed there has only ever been one laboratory detailing convincing myelin formation in dissociated mouse myelinating SC-DRG neuron cocultures, however this was never published as a step by step detailed protocol (Stevens et al, 1998; Stevens & Fields, 2000). In the last twenty years, there have been no published studies demonstrating myelination in fully dissociated mouse SC-mouse DRG cocultures. This has largely prevented the use of cells, particularly SCs, from transgenic mice in cocultures and thus restricted the ability to study SC-axon interactions in a system that can be readily manipulated and live imaged and results directly applied back to in vivo findings in the same species.

© 2023. Published by The Company of Biologists under the terms of the Creative Commons Attribution License (https://creativecommons.org/licenses/by/4.0/).

Reviewer 1. The use of embryonic DRG neurons or SC isolated from P2 animals are arguably physiologically not the same cells that are affected by traumatic nerve injury, which happen most often than not in adult. This is a problem in the long-term reliance on this approach to study axotomy peripheral nerve regeneration.

Author response: We agree with the reviewer that one should always be cautious with the use of embryonic/neonatal cells to directly refer them to adult cellular mechanisms. We have added discussion of this point to the discussion:

Discussion.
One limitation of our coculture model and indeed all coculture and cell culture models that are used to investigate cellular and molecular mechanisms in nerve injury is that the cells are obtained from embryonic or neonatal animals. This is an important caveat when applying results from cell culture to adult in vivo nerve injury. However, while we would argue that cell culture approaches should always be used in combination with in vivo study it is important to remember that nerve injury is not restricted to adults and brachial plexus injury secondary to birth trauma is unfortunately a significant clinical problem (Pondaag et al, 2007). Furthermore, neonatal SCs replicate many of key cellular and molecular mechanisms seen in adult SCs after injury, including JUN upregulation, myelinophagy, promotion of axon growth and expression of key repair program transcripts (Arthur-Farraj et al, 2012; Gomez-Sanchez et al, 2015; Arthur-Farraj et al, 2017; Parkinson et al, 2008). A future development would be to try to adapt this protocol to make a coculture model with adult mouse or even human cells.

Author response: Additionally, we already know Schwann cells in P5 neonatal mice in vivo after nerve transection demyelinate in a similar way to Schwann cells in adult mice and that neonatal cells in vivo and in vitro require the transcription factor c-Jun to do so (Parkinson et al., 2008 JCB Fig.7).

Moderate points:
Reviewer 1"there are no established protocols in the field describing the use of mouse SCs with mouse DRG neurons in dissociated myelinating cocultures". The use of mouse cells is laudable, but it is not necessarily a technical innovation, or at least the current manuscript does not explain why their approach particularly suitable to mouse Schwann cells.

Author response: We feel that a detailed working protocol for compartmentalised dissociated mouse myelinating cocultures showing convincing and extensive myelination has been missing from our field for a long time. We agree that it is an incremental technical advance, but it is an important one. We have modified the title as we explained above. We have explained this point more clearly in the introduction, results, and discussion with the following additions:

Introduction.
Indeed there has only ever been one laboratory detailing convincing myelin formation in dissociated mouse myelinating SC-DRG neuron cocultures, however this was never published as a step by step detailed protocol (Stevens et al, 1998; Stevens & Fields, 2000). In the last twenty years, there have been no published studies demonstrating myelination in fully dissociated mouse SC-mouse DRG cocultures. This has largely prevented the use of cells, particularly SCs, from transgenic mice in cocultures and thus restricted the ability to study SC-axon interactions in a system that can be readily manipulated and live imaged and results directly applied back to in vivo findings in the same species.

The consensus within the field is that inducing myelination in dissociated mouse SCs is challenging. Certainly, induction of myelin differentiation with cyclic adenosine monophosphate (cAMP) analogues or elevating agents, such as forskolin, is more difficult in mouse SC monocultures compared to rat SC cultures. This is because mouse SCs require additional exogenous β-neuregulin-1 (βNRG1), plating on poly-L-lysine (PLL) instead of poly-D-lysine (PDL), and low concentration horse serum as opposed to foetal calf serum (Stevens et al, 1998; Arthur-Farraj et al, 2011; Päiväläinen et al, 2008).

© 2023. Published by The Company of Biologists under the terms of the Creative Commons Attribution License (https://creativecommons.org/licenses/by/4.0/).

Author response: We have now explained more clearly that without plating on Matrigel and the regular addition of Matrigel to the myelination medium that mouse cocultures do not myelinate with ascorbic acid or indeed addition of NRG1 nor forskolin. Please see NEW DATA in Supplemental figure 1. We have added the following paragraph to the results section.

Results
Importantly, we found that L-ascorbic acid was insufficient to induce substantial myelination in our cultures, unlike in rat SC-DRG cocultures, and in the one previously published dissociated mouse SC-DRG protocol (Stevens et al, 1998). In fact, plating cocultures on laminin, adding ascorbic acid (50 μg ml−1), βNRG1 (10 ng ml−1) and forskolin (10 μM) induced very few myelin sheaths (Supp. Fig. 1). Only when cultures were plated on Matrigel® and further Matrigel® was added to the myelination medium for each medium change, were we able to visualise robust reproducible myelination in our cocultures (Supp. Fig. 1).

Author response: We have also added further discussion on how our protocol differs from the Stevens 1998 and other protocols in the discussion.

Discussion
Our protocol differs somewhat from the one used by Stevens et al., 1998 to induce myelination in dissociated mouse SC-DRG cocultures, as they used ascorbic acid and 10% horse serum and presumably plated their cultures on laminin, though they do not explicitly detail this (Stevens et al, 1998). In our preliminary experiments we were unable to visualise much myelination with use of laminin, ascorbic acid or indeed if βNRG1 and high concentration forskolin was added to the medium for up to four weeks. However, if we plated cocultures on Matrigel® and continuously added it to the myelination medium then we saw comparable levels of myelination in our mouse cocultures to that of rat cocultures (Eldridge et al, 1987). This approach of using Matrigel® to enhance myelination has previously been successfully employed in cultures of human iPSC sensory neurons with rat SCs and in non-dissociated mouse DRG explant cultures (Clark et al, 2017; Päiväläinen et al, 2008). Importantly, we used growth factor depleted Matrigel® as standard Matrigel® preparations contain substantial amounts of Transforming growth factor β (TGFβ) which is a known inhibitor of myelination (Einheber et al, 1995). Additionally, the majority of rat and mouse coculture protocols plate cells on glass whereas we found cultures were healthier and myelinated better when cultured on plastic Alcar® coverslips.

Discussion
Furthermore, as this is a dissociated and compartmentalised purely mouse cell culture system, one can utilise the vast array of transgenic and knockout lines available to study neuron-SC interactions in more detail, without concern of contaminating endogenous SCs and other non-neuronal cells that remains a drawback of current mouse dissociated or non-dissociated DRG explant models.

Reviewer 1: The figures in the paper are largely descriptive. They are very little quantitative measurement. Thus, the readers will have a hard to determine, if they replicate the proposed approach, whether their efficient is on par with the current authors.

Author response: We have now added quantification of myelin segments per mm2, percentage of SCs that myelinate and quantification of the interperiodic distance of the myelin formed. This is all included in a new version of TABLE 1. We have discussed this data in the results section as follows.

Results
Quantifying the number of PRX positive myelin segments we found that there were 325.33 ± 12.3 sheaths per mm2, comparable to what has been originally described in rat SC/DRG cocultures and two fold more extensive myelination than recently described compartmentalised rat cocultures models (n=3; Table 1; Eldridge et al, 1987; Vaquié et al, 2019). Furthermore, 25.47 ± 1% of Schwann cells were myelinating in our cultures (n=3; Table 1). To confirm that cocultured myelinated SCs formed compact myelin we performed electron microscopy (EM), which revealed compact myelin formation with multiple myelin wraps and formation of readily visible major dense (MDL) and intraperiod lines (IPL; Fig. 2D). Additionally, we measured the periodicity, i.e., the distance between two adjacent major dense lines, to make sure myelin was compacted.

© 2023. Published by The Company of Biologists under the terms of the Creative Commons Attribution License (https://creativecommons.org/licenses/by/4.0/).

Interperiodic distance was 12.16 ± 0.28 nm, in line with previous reports (n=3; Table 1; Boutary et al, 2021; Fernando et al, 2016; García-Mateo et al, 2018; Giese et al, 1992; Perrot et al, 2007).

Author response: Interestingly, after we performed this analysis, we realised we have double the level of myelination in our mouse cultures (325.33 ± 12.3 per mm2) than in the compartmentalised myelinating rat cocultures in Vaquie et al., 2019 (147 ± 27 internodes per mm2 (n = 3)).

Author response: We have also quantified the JUN upregulation after injury in both myelinating and aligned cocultures. See Fig. 3B-E).

Results
Additionally, we noted a strong upregulation of JUN protein in SCs 12 hours after axotomy (Fig. 3B and C). We also saw significant JUN upregulation 12 hours after axotomy in cocultures with aligned SCs (Fig. 3D and E).

Author response: The above quantification is in addition to the quantification of the rate of axon degeneration in the presence and absence of aligned and myelinating Schwann cells in Fig. 4B. We have also quantified the % of Schwann cells that contained axonal debris after injury – this data is now quoted in the text as we removed Fig. 4E.

We thank the reviewer for asking for additional quantification as this has improved the manuscript.

Minor points:
Fig.4B and E should show individual data points.

Author response: We have added the individual data points to Fig.4B. We have removed Fig.4E and instead quoted the data in the results section as follows:

Results
When we quantified this phenomenon, we found that 97.84 ± 1.462% (n=2) of SCs in our cocultures contained mCherry-labelled axonal fragments.

Reviewer #1 (Significance (Required)):

In addition, to the demonstration of feasibility of this in vitro approach, the main finding by the authors is that SCs have a role in neuronal protection and support is key for peripheral nerve regeneration. Thus while in vitro approach does not add key information that do not already exists in the field, it somewhat confirms that the effect is SC autonomous. Overall the approach is interesting and has potential, but the study currently lack a demonstration of its usefulness to the community.

It would have been interesting to have the authors discuss the advantages of their approach in comparison to other innovative approaches to study SC-axon interactions that have been developed in the last decade (i.e., 3D environment, microfluidic approach, transwell systems). There is also a lack of citations about similar studies in the field.

Author response: We direct the reviewer to the following paragraphs in the introduction and the discussion, which we have now elaborated on further post peer review. We discuss all relevant cocultures studies in mouse as well as all the relevant microfluidic studies and 3D coculture studies as well as the one human nerve organoid study. We found two additional studies, Numata-Uematasu 2023 using DRG mouse explant cultures and Park et al., 2021 using motorneuron-SC cocultures which we have now added to the discussion. We also briefly discuss transwell studies to assess migration as the reviewer requested.

We also discuss in detail the two microfluidic coculture injury studies Babetto et al., 2020 and Vaqiue et al., 2019 extensively throughout the manuscript. We have added further discussion of the similarities and differences between theirs and our approach in the discussion.

© 2023. Published by The Company of Biologists under the terms of the Creative Commons Attribution License (https://creativecommons.org/licenses/by/4.0/).

Introduction.
Protocols exist where endogenous mouse SCs are used to myelinate dissociated or non-dissociated DRG explant cultures. (Shen et al, 2014; Harty et al, 2019; Sundaram et al, 2021; Stettner et al, 2013; Numata-Uematasu et al, 2023). Furthermore, another protocol seeded exogenous SCs onto non-dissociated DRG explant cultures (Päiväläinen et al, 2008). Other laboratories seed cultured rat SCs onto dissociated mouse DRG axons (Taveggia & Bolino, 2018). Use of dissociated or non-dissociated DRG explants cultures preclude many experimental uses, such as using SCs from different transgenic animals and separate transfection of SCs and neurons with viruses for live imaging or genetic manipulation, and easy use of microfluidic chambers to allow injury studies and separate drug treatments to neurons or SCs. The reason for this is that antimitotics cannot be used in dissociated or non-dissociated DRG explant cultures as this depletes SCs, and the culture quickly becomes contaminated with other non-neuronal cell types, such as satellite cells and fibroblasts migrating out of the DRG. Furthermore, use of exogenous SCs in a non-dissociated DRG explant culture risks, after a period of antimitotic exposure, which was developed by Päiväläinen et al., 2008 still risks potential contamination from endogenous SCs and satellite glia migrating out of the DRG explant over time. This occurs because antimitotic treatment is unlikely to fully penetrate the whole DRG without prior dissociation. Additionally, a compartmentalised culture system cannot be readily used with non-dissociated DRG explant cultures (Päiväläinen et al, 2008).

Discussion.
Furthermore, our protocol is complementary to the recently described 3D mouse myelinating SC-motor neuron coculture system using collagen hydrogels (Hyung et al, 2021; Park et al, 2021). It will be interesting in the future to up titrate the concentration of Matrigel®, which is similar to collagen hydrogels, in our cultures to see whether further increasing extracellular matrix viscosity and stiffness improves our myelination efficiency even further. While it is possible to study cell migration in microfluidic cell culture devices, transwell models offer significant advantages to study this cellular phenomenon (Negro et al, 2022). To date, there have been no published studies of successful myelination in human SC-neuron coculture systems. Despite this rat SCs have been shown to readily myelinate human-induced pluripotent stem cell (iPSC)-derived sensory neurons and an iPSC-derived peripheral nerve organoid system which does contain myelinating SCs has recently been described (Clark et al, 2017; Van Lent et al, 2022).

These findings confirm the observations of both Babetto et al., 2020 and Vaquié et al., 2019 who used rat SCs in similar microfluidic culture systems (Vaquié et al, 2019; Babetto et al, 2020). We have shown that the axo-protective observation seen by Babetto et al., 2020 does not rely on myelination status, which was an outstanding question from that study (Babetto et al, 2020). Furthermore, in an advance from previous studies, we have visualised the axo-protective and axon clearance phenomena in the same culture and shown that they are temporally separated, with axon fragmentation and debris clearance by SCs occurring at much later timepoints after axotomy. Several different culture and experimental conditions preclude direct comparison of our study with both those of Vaquié et al., 2019 and Babetto et al., 2020. These include the use of rat SCs in both prior studies and that Babetto et al., 2020 mixed rat SC with mouse DRG axons; length of time in culture, time points quantified after injury and distance from injury and site of analysis. Babetto et al., 2020 performed axotomy on relatively short term cocultures (6 days in vitro) whereas Vaiquié et al., 2019 cultured for at least 4 weeks and in our case 6 weeks prior to axotomy. Vaiquié et al., 2019 removed nerve growth factor (NGF) prior to laser axotomy and quantified proximally (though also imaged distally) whereas both our study and Babetto et al., 2020 kept NGF in the medium, performed axotomy with a scalpel and quantified more distally and in our case extremely distal, where only individual neurites and no axon bundles were visible. Vaquié et al., 2019 had SCs on both sides of the barrier in the microfluidic chambers whereas we seeded SCs only in the axonal/bottom compartment. Additionally, our cocultures had both forskolin and βNRG1 added to help induce myelination, whereas these factors are not required in rat myelinating cocultures. Finally, it is important to permeabilise myelinated cultures with acetone after fixation, as we did, to visualise the entire axon through heavily myelinated segments otherwise axon integrity cannot be reliably assessed in a quantitative manner (Vaquié et al, 2019; Babetto et al, 2020).

Because of the lack of key novel mechanisms, and lack of discussion on what this approach is superior to others in vitro approach limits the impact of the study and the excitement of the reader, even from the SC-axon community.

© 2023. Published by The Company of Biologists under the terms of the Creative Commons Attribution License (https://creativecommons.org/licenses/by/4.0/).

Author response: We have developed the first compartmentalised fully dissociated mouse myelinating coculture system in over twenty years. Thanks to the reviewer's suggestions, we have now shown that myelination is comparable to the original rat cocultures from the Bunge lab, which is the gold standard in the field, and superior to recently described compartmentalised rat coculture system by Vaquie et al., 2019. We have provided a detailed step by step protocol to allow other researchers to use our technique.

Additionally, thanks to the reviewer, we have now described in detail exactly how our protocol differs from others and why we succeeded to get mouse SCs to myelinate so robustly in a fully dissociated coculture (see previous answers). This is an incremental but important advance given that studies currently use a coculture system using entirely cells from rat, or where rat Schwann cells are seeded on mouse axons, or dissociated or non-dissociated mouse explant cultures are used which abrogates using neurons and SCs from different transgenic mice.

As this is a methods paper, we did not intend to describe novel molecular mechanisms though our method is already being used for such purposes by ourselves and a collaborator as outlined above in prior answers. We did not make this clear and we hope the extensive revision of the manuscript now addresses this point. Despite this, we have shown that the axo-protective effect of SCs is independent of myelination status, which is an advance on what is known in the literature.

Finally, in addition to myelination, we have demonstrated that one can study all the key components of the SC and axonal response to injury in a quantifiable way in addition to demonstrating that these cocultures can be live imaged and used for drug studies. None of the prior mouse studies looked at injury responses of axons nor SCs. We believe this will be of use to the community.

Reviewer #2 (Evidence, reproducibility and clarity (Required)):

In the manuscript entitled "Distinct axo-protective and axo-destructive roles for Schwann cells after injury in a novel compartmentalised mouse myelinating coculture system", Arthur-Farraj and colleagues detail a method of dissociated coculture of mouse-DRG neurons and Schwann cells in microfluidic chambers. In this system, neurons and Schwann cells harvested from the same or from different animals are grown in different compartments that are connected by microgrooves, thereby allowing for spatial and diffusive separation. Neurons are shown to extent their axons across the microgroove barrier to the glial compartment where Schwann cells align with the axons and myelination can be induced. Detailed analysis of myelination and axon injury/degradation are presented as use cases, including the capability to genetically and pharmacologically manipulate neurons and Schwann cells independently, which also enabled fluorescent life cell imaging. The authors then examine the effect of immature/premyelinating and myelinating Schwann cells on the rate of axon degeneration. Upon axotomy Schwann cells significantly delayed degeneration, with no difference between non-myelinating and myelinating Schwann cells. Finally, live imaging during axon degeneration with fluorescent proteins separately expressed in neurons and Schwann cells demonstrated that Schwann cells ingest axonal fragments.

Major comment:
In establishing a much needed in-vitro system for PNS myelination and injury research the paper represents a valuable contribution to the PNS community. However, I find the presentation of aspects concerning a protective/destructive role of Schwann cells somewhat inconclusive. That these roles exist has been known, as the authors discuss. Then what does this study contribute concerning the open question that was raised by the discrepancies between Babetto et al., 2020 and Vaquié et al., 2019, i.e. how Schwann cells contribute to axon survival/regeneration after injury? Essentially, the only significant conclusion in this regard is that myelinating and non-myelinating mouse Schwann cells do not differ in their capability to protect axons from degeneration. The manuscript, including the title, would benefit from focusing more on this aspect. In particular, the discussion of the factors that lead to the still remaining apparent discrepancies between Babetto et al., 2020 and Vaquié et al., 2019 and this study should be revised. The authors state that "The study by Vaiquié et al., 2019 quantified axon fragmentation proximally in the microgrooves at timepoints starting from 12 hours after axotomy." (Discussion). While this observation is accurate, Jacob and colleagues also show accelerated, obviously distal axon degeneration in the presence of Schwann cells (Figure 3C in Vaquié et al., 2019). It is therefore

© 2023. Published by The Company of Biologists under the terms of the Creative Commons Attribution License (https://creativecommons.org/licenses/by/4.0/).

unlikely that the discrepancies stem from analysis of more proximal vs. more distal axons, or the timepoints of analyses. In my opinion, a further study (using the coculture system presented in this manuscript) that compares the role of Schwann cells from rats and mice, and that includes analysis of more proximal and distal axon degeneration as well analysis of axon regeneration is needed. In a rework of the manuscript, the authors may therefore elaborate more on the shortcomings of the present study, or alternatively soften the aims of the study in the first place.

Author response: We thank the reviewer for their comments, and we agree that the title and the aims of the manuscript were confusing. We didn't make it explicit that this was a methods paper, and that we didn't intend to show entirely novel findings but instead thoroughly characterise our mouse system so that it is comparable to what has been done for rat cocultures. We have now made this clearer by removing the word novel in the title and changing the title to the following:

A method for mouse myelinating Schwann cell-DRG neuron compartmentalised cocultures: myelination status does not influence the axo-protective effect of Schwann cells after injury.

Author response: We have decided to focus the manuscript more on the comparison of myelinating versus non-myelinating cocultures, given that we have shown that the axo-protective effect of SCs is independent of myelination status, which is an advance on what is known in the literature. We have added further characterisation of our aligned cocultures with p75NTR immuno and EM images (Fig.1D and E). We have added the following summary of how our study relates to findings of Babetto et al., 2020 and Vaquie et al., 2019 in the discussion, in line with the reviewer's suggestions.

Discussion.
These findings confirm the observations of both Babetto et al., 2020 and Vaquié et al., 2019 who used rat SCs in similar microfluidic culture systems (Vaquié et al, 2019; Babetto et al, 2020). We have shown that the axo-protective observation seen by Babetto et al., 2020 does not rely on myelination status, which was an outstanding question from that study (Babetto et al, 2020). Furthermore, in an advance from previous studies, we have visualised the axo-protective and axon clearance phenomena in the same culture and shown that they are temporally separated, with axon fragmentation and debris clearance by SCs occurring at much later timepoints after axotomy.

Author response: We have now added more in-depth discussion of the similarities and differences between Babetto et al., 2020 and Vaquie et al., 2019 and our approach in the discussion.

Discussion.
Several different culture and experimental conditions preclude direct comparison of our study with both those of Vaquié et al., 2019 and Babetto et al., 2020. These include the use of rat SCs in both prior studies and that Babetto et al., 2020 mixed rat SC with mouse DRG axons; length of time in culture, time points quantified after injury and distance from injury and site of analysis. Babetto et al., 2020 performed axotomy on relatively short term cocultures (6 days in vitro) whereas Vaiquié et al., 2019 cultured for at least 4 weeks and in our case 6 weeks prior to axotomy. Vaiquié et al., 2019 removed nerve growth factor (NGF) prior to laser axotomy and quantified proximally (though also imaged distally) whereas both our study and Babetto et al., 2020 kept NGF in the medium, performed axotomy with a scalpel and quantified more distally and in our case extremely distal, where only individual neurites and no axon bundles were visible. Vaquié et al., 2019 had SCs on both sides of the barrier in the microfluidic chambers whereas we seeded SCs only in the axonal/bottom compartment. Additionally, our cocultures had both forskolin and βNRG1 added to help induce myelination, whereas these factors are not required in rat myelinating cocultures. Finally, it is important to permeabilise myelinated cultures with acetone after fixation, as we did, to visualise the entire axon through heavily myelinated segments otherwise axon integrity cannot be reliably assessed in a quantitative manner (Vaquié et al, 2019; Babetto et al, 2020).

Author response: We would like to add that we showed Claire Jacob, senior author of the Vaquie et al., 2019 study, our manuscript prior to peer review and she offered helpful comments, which we incorporated into the manuscript, which is why she is acknowledged. We have also discussed our findings with Elisabetta Babetto as well.

© 2023. Published by The Company of Biologists under the terms of the Creative Commons Attribution License (https://creativecommons.org/licenses/by/4.0/).

While it would be a great future study to compare both axon degeneration rates in rat and mouse cocultures this was not the original intention of our study. We believe we have included enough detail of our experimental procedures, including the distance from the barrier we imaged axon degeneration, a crucial bit of information missing from the other studies, should others want to perform a comparative study between rats and mice.

Methods
Five images at a distance of between 1.2-1.4mm from the microgroove barrier (the most distal part of the culture that could be imaged) were quantified per culture, taken in comparable locations in each culture.

Author response: I would add that one of our previous studies (Arthur-Farraj et al., 2012 Neuron, Fig5I) has already looked at axon outgrowth/regeneration in dissociated non-myelinating mouse SC-DRG co-cultures. We showed the presence of Schwann cells accelerates axon regeneration/outgrowth and this relies upon Schwann cell c-JUN.

We have now added quantification of the extent of myelination in our cocultures and it is comparable to the original Bunge lab rat cocultures and more extensive than the Vaquie et al., 2019 coculture.

Results
Quantifying the number of PRX positive myelin segments we found that there were 325.33 ± 12.3 sheaths per mm2, comparable to what has been originally described in rat SC/DRG cocultures and two fold more extensive myelination than recently described compartmentalised rat cocultures models (n=3; Table 1; Eldridge et al, 1987; Vaquié et al, 2019). Furthermore, 25.47 ± 1% of Schwann cells were myelinating in our cultures (n=3; Table 1).

Methods
To quantify the number of myelin segments per area, we counted the number of myelin segments for five areas per culture for three cultures and normalised this per mm2. To quantify the percentage of Schwann cells in myelinating cocultures that are actively myelinating, we quantified the number of myelin segments and the number of DAPI-positive nuclei for five areas per culture for three cultures.

Minor comments:
- Figure 2D: From the electron micrograph there is no doubt that compact myelin is formed, however to me it seems the compaction is not complete. A rough estimation with the aid of the provided scale bar resulted in an interperiodic distance of about 17 nm, which contrasts with the remarkably well reproduced values reported in multiple reports using conventional specimen preparation (like in this study), of which I am citing just a few: about 13 nm in rat ex vivo nerve (Peterson and Pease, J. Ultrastruct Res 1972; Fledrich et al., Nat Commun 2018), 12 nm (Giese et al., Cell 1992), 12.2 nm (Perot et al., J Neurosci 2007), about 12 (Fernando et al., Nat Commun 2016), about 13 nm (García-Mateo et al., Glia 2017) or about 13 nm in mouse ex vivo (Boutary et al., Commun Biol 2021), which was also reproduced with about 13 nm in rat in vitro (Taveggia and Bolino, Methods Mol Biol 2018). The authors should acknowledge this deviation and might discuss possible reasons.

Author response: We have now provided a more representative EM image of our myelination (Fig. 2D). Additionally, thanks to the reviewer's comments we have now quantified the interperiodic distance and find it is comparable to the studies the reviewer suggested. We have added the data to the new Table 1 and added the references the reviewer advised. Please see the additions to the methods and the results section regarding this new data below.

Results
To confirm that cocultured myelinated SCs formed compact myelin we performed electron microscopy (EM), which revealed compact myelin formation with multiple myelin wraps and formation of readily visible major dense (MDL) and intraperiod lines (IPL; Fig. 2D). Additionally, we measured the periodicity, i.e., the distance between two adjacent major dense lines, to make sure myelin was compacted. Interperiodic distance was 12.16 ± 0.28 nm, in line with previous reports

© 2023. Published by The Company of Biologists under the terms of the Creative Commons Attribution License (https://creativecommons.org/licenses/by/4.0/).

(n=3; Table 1; Boutary et al, 2021; Fernando et al, 2016; García-Mateo et al, 2018; Giese et al, 1992; Perrot et al, 2007).

Methods
To measure interperiodic distance, we measured at least 10 periods per myelinated fibre for at least three fibres per sample for three separate samples.

- Figure 4A,B: The result of a slowed axon degeneration in coculture relies on the accurate assessment of continuity of the NFL staining. While the authors report that acetone permeabilization was necessary to afford complete penetration of the used antibodies in myelinating cultures, I cannot see why the authors have not used the same staining protocol for all cultures, as it is detailed in the method section. While I consider it unlikely that the staining conditions have led to an apparent delay of degeneration in coculture, experiments should generally be performed under identical conditions, unless there are good reasons not to do so. If this is not the case, it will be reassuring to see the same effect when identical staining conditions are employed. On the same note, do the compared cultures have the same age, i.e. have the neuron monocultures been in vitro for the same time as the cocultures?

Author response: We thank the reviewer for picking up this inaccuracy in the manuscript. We can confirm that for the purposes of the axon degeneration experiment all cultures were stained using exactly the same staining protocol. Additionally, we were very careful to maintain all cultures for exactly the same time in culture – 6 weeks. Additionally, axon only cultures were maintained in myelination medium to make sure medium constituents were not responsible for the observed differences in degeneration rate. We have added the following elaboration to the methods section to clarify these points.

Methods
Axon only cultures related to Figure 1 were permeabilised in PBS + 0.5% Triton (Merck) + 5% HS + 5% donkey serum (DS, Merck - D9663) at RT for 1 hour. For the purposes of quantifying the rate of axon degeneration (Figure 4) both axon only cultures and cocultures with SCs were permeabilised in 50% Acetone for 2 minutes, 100% Acetone for 2 minutes, 50% Acetone for 2 minutes (all at RT), and then blocked in PBS + 0.5% Triton + 5% HS + 5% DS at RT for 1 hour.

Methods
All cultures (axon only, aligned SCs and myelinating SCs) were cultured for 6 weeks prior to axotomy experiments. To minimise the possibility that medium constituents were responsible for differences in axon degeneration rates, axonal compartments of axon only cultures were cultured in medium containing 10 ng ml-1 βNRG1 and 10 μM forskolin (axon only medium, extended methods section D6) once SCs were seeded on other cultures, and then switched into myelination medium (additional Matrigel® and 50 μg ml−1 L-Ascorbic Acid), 24 hours before axotomy. Bottom compartments of aligned SC cultures, 24 hours before axotomy, were switched into DRG/SC medium containing 10 ng ml-1 βNRG-1, 10 μM forskolin and 50 μg ml−1 L-Ascorbic Acid, which is insufficient to induce myelination in mouse cultures. Bottom compartments of myelinating cocultures were medium changed into fresh myelination medium (Extended methods section D7) 24 hours prior to axotomy.

- In several instances of the manuscript, the term "transfection" is used to refer to lentiviral gene transfer. I advise to use the more appropriate term "transduction" instead
- I could not seem to find a meaningful reference to the microfluidic chambers that were used in the study. The protocol should contain details on the device and source of supply in order to enable potential readers to execute the protocol

Author response: We thank the reviewer for this comment. We have replaced transfection with transduction throughout the manuscript. Please see the track changes manuscript for all instances.

Reviewer #2 (Significance (Required)):

The paper presents a convincing establishment of a dissociated coculture derived exclusively from mouse that leads to robust myelination. As the manuscript correctly states, Schwann cell culture

© 2023. Published by The Company of Biologists under the terms of the Creative Commons Attribution License (https://creativecommons.org/licenses/by/4.0/).

and especially coculture with neurons has been experienced difficult in the field, and by providing a detailed protocol as well as demonstrating how the coculture system can be used to address important questions of PNS myelination and repair, the paper fills an important gap. However, the experiments directed to the role of Schwann cells in axon degeneration do not clarify much, which should be better addressed in the discussion and also by modifying the title accordingly.
The paper will be of high value for basic researchers that are interested in performing studies addressing cellular and molecular mechanisms of myelination and repair in the PNS. Importantly, the paper can pave the way to usage of transgenic or knockout mouse models in coculture. Thereby it might spark interest also in those researchers that use transgenic and knockout mouse models and who have so far refrained from using coculture models.

Field of expertise of the reviewer: Cellular and molecular mechanisms of myelination and growth signaling in the PNS; in-depth experience with DRG coculture models from rats and mice

Author response: We thank the reviewer for their kind comments. We have now modified the title, aims and discussion of the manuscript in line with the reviewer's suggestions.

Reviewer #3 (Evidence, reproducibility and clarity (Required)):

SUMMARY
The authors present a detailed protocol for co-cultures of mouse DRGs with mouse SCs using microfluidics. In this model, cells of interest grow in different compartments while allowing for axons to grow in between, thereby making them accessible to injury induction. Using this experimental system, the authors show that myelination occurs, myelin gets compacted and acquires nodal organization. The authors then show that such a system allows for compartment-specific lentivirus transduction and live imaging. Next, they perform physical and chemical axonal injury and show that at early time point pos- injury the presence of SCs protects from axonal degeneration regardless of the myelination status, and helps with clearing of damaged axons at later time points.
Major comments:

The novelty of the study is questionable.
While the model is well described and appears to be useful for the proposed applications (live imaging, transduction, injury model), the arguments provided regarding its novelty are not fully convincing. The main argument from the authors of this paper is that there are no established protocols describing the use of mouse SCs with mouse DRG neurons in dissociated myelinating cocultures. However, this appears to be inaccurate, as the model described in Stevens et al., 1998 (cited in the paper) uses mouse DRG neurons dissected at E13.5 with mouse SCs dissected at P3 to study myelination. Also, in Päiväläinen et al., 2008, mouse DRGs and SCs are cultured from transgenic mice at different developmental ages, thereby arguing that coculture models have been previously successfully implemented. The main difference appears to be rather the compartmentalization of SCs and DRGs which appears to be a mouse adaptation of the rat model described by Vaquie et al,2019. Based on the above, it seems imperative for the authors to tone down the novelty aspect and provide a more thorough discussion on how the current novel differs from protocols in published study, highlighting advantages and caveats for each.

Author response: We agree with the reviewer that we did not make the case clear enough for how our coculture model adds to what is currently described in the literature. We have now changed the title and removed the word novel. New title:

A method for mouse myelinating Schwann cell-DRG neuron compartmentalised cocultures: myelination status does not influence the axo-protective effect of Schwann cells after injury.

Author response: We have now added the following paragraph to the introduction:

Introduction.
Indeed there has only ever been one laboratory detailing convincing myelin formation in dissociated mouse myelinating SC-DRG neuron cocultures, however this was never published as a step by step detailed protocol (Stevens et al, 1998; Stevens & Fields, 2000). In the last twenty years, there have been no published studies demonstrating myelination in fully dissociated mouse SC-mouse DRG

© 2023. Published by The Company of Biologists under the terms of the Creative Commons Attribution License (https://creativecommons.org/licenses/by/4.0/).

cocultures. This has largely prevented the use of cells, particularly SCs, from transgenic mice in cocultures and thus restricted the ability to study SC-axon interactions in a system that can be readily manipulated and live imaged and results directly applied back to in vivo findings in the same species.

Author response: Regarding the study by Päiväläinen et al, 2008, they did not fully dissociate their DRGs (see Fig.1 which demonstrates a DRG explant) and thus it is a non-dissociated DRG explant model. While they demonstrated convincing myelination due to the use of Matrigel which we acknowledge them for, their model is not perfectly suited for the use of neurons and SCs from different transgenic animals as the use of a DRG explant, even with temporary use of an antimitotic, risks contamination by endogenous SCs and satellite glia over time, especially as their model is not compartmentalised. We discuss the caveats of their protocol and those using dissociated mouse explant cocultures in a revised paragraph in the introduction.

Introduction.
Protocols exist where endogenous mouse SCs are used to myelinate dissociated or non-dissociated DRG explant cultures. (Shen et al, 2014; Harty et al, 2019; Sundaram et al, 2021; Stettner et al, 2013; Numata-Uematasu et al, 2023). Furthermore, another protocol seeded exogenous SCs onto non-dissociated DRG explant cultures (Päiväläinen et al, 2008). Other laboratories seed cultured rat SCs onto dissociated mouse DRG axons (Taveggia & Bolino, 2018). Use of dissociated or non-dissociated DRG explants cultures precludes many experimental uses, such as using SCs from different transgenic animals and separate transfection of SCs and neurons with viruses for live imaging or genetic manipulation, and easy use of microfluidic chambers to allow injury studies and separate drug treatments to neurons or SCs. The reason for this is that antimitotics cannot be used in dissociated or non-dissociated DRG explant cultures as this depletes SCs, and the culture quickly becomes contaminated with other non-neuronal cell types, such as satellite cells and fibroblasts migrating out of the DRG. Furthermore, use of exogenous SCs in a non-dissociated DRG explant culture risks, after a period of antimitotic exposure, which was developed by Päiväläinen et al., 2008 still risks potential contamination from endogenous SCs and satellite glia migrating out of the DRG explant over time. This occurs because antimitotic treatment is unlikely to fully penetrate the whole DRG without prior dissociation. Additionally, a compartmentalised culture system cannot be readily used with non-dissociated DRG explant cultures (Päiväläinen et al, 2008).

Author response: We have also added further discussion on how our protocol differs from the Stevens 1998 and other protocols in the discussion.

Discussion
Our protocol differs somewhat from the one used by Stevens et al., 1998 to induce myelination in dissociated mouse SC-DRG cocultures, as they used ascorbic acid and 10% horse serum and presumably plated their cultures on laminin, though they do not explicitly detail this (Stevens et al, 1998). In our preliminary experiments we were unable to visualise much myelination with use of laminin, ascorbic acid or indeed if βNRG1 and high concentration forskolin was added to the medium for up to four weeks. However, if we plated cocultures on Matrigel® and continuously added it to the myelination medium then we saw comparable levels of myelination in our mouse cocultures to that of rat cocultures (Eldridge et al, 1987). This approach of using Matrigel® to enhance myelination has previously been successfully employed in cultures of human iPSC sensory neurons with rat SCs and in in non-dissociated mouse DRG explant cultures (Clark et al, 2017; Päiväläinen et al, 2008). Importantly, we used growth factor depleted Matrigel® as standard Matrigel® preparations contain substantial amounts of Transforming growth factor β (TGF β) which is a known inhibitor of myelination (Einheber et al, 1995). Additionally, the majority of rat and mouse coculture protocols plate cells on glass whereas we found cultures were healthier and myelinated better when cultured on plastic Alcar® coverslips.

Author response: We have added further discussion of comparable models in the literature in the discussion.

Discussion.
Furthermore, our protocol is complementary to the recently described 3D mouse myelinating SC-motor neuron coculture system using collagen hydrogels (Hyung et al, 2021; Park et al, 2021). It

© 2023. Published by The Company of Biologists under the terms of the Creative Commons Attribution License (https://creativecommons.org/licenses/by/4.0/).

will be interesting in the future to up titrate the concentration of Matrigel®, which is similar to collagen hydrogels, in our cultures to see whether further increasing extracellular matrix viscosity and stiffness improves our myelination efficiency even further. While it is possible to study cell migration in microfluidic cell culture devices, transwell models offer significant advantages to study this cellular phenomenon (Negro et al, 2022). To date, there have been no published studies of successful myelination in human SC-neuron coculture systems. Despite this rat SCs have been shown to readily myelinate human-induced pluripotent stem cell (iPSC)-derived sensory neurons and an iPSC-derived peripheral nerve organoid system which does contain myelinating SCs has recently been described (Clark et al, 2017; Van Lent et al, 2022).

Next, the authors emphasize the conflicting results of two articles, Babetto et al., 2020 and Vaquie et al., 2019, as the basis to use their newly developed model in the same species and testing two ages corresponding to distinct myelination states. However, both studies reach the same conclusion as the current study, that SCs have a protective role, although at two different developmental time points. As such, it is likely that multiple mechanisms may account for the protective effect of SC on axonal damage, and therefore the different studies do not seem conflicting but rather complementary. Yet, it is interesting that this manuscript shows that the myelination status of SCs does not impact their ability to slow down degeneration and yet it confirms that different timing after injury elicits different behaviors in SCs, as suggested by the studies of Babetto et al., 2020 and Vaquie et al., 2019. In other words, a more accurate description of the results of these two studies is needed and a better explanation of what the authors consider to be conflicting and why (there could be more differences than species and myelination, for instance, such as the method used for axotomy - laser vs cut with scalpel which tear and pull membranes).

Author response: We would like to humbly correct the reviewer that the studies by Babetto et al., 2020 and Vaquie et al., 2019 do not reach the same conclusion that Schwann cells have a protective role. Instead, they describe axon protection (Babetto et al., 2020) and axon fragmentation (Vaquie et al., 2019). Our studies now visualise both phenomena in the same culture system. We have now made this point more explicit as well as highlighted the one conceptual advance our methods paper makes on the current literature, which is that myelination status does not influence the SC axo-protection, as the reviewer suggested.

Discussion.
These findings confirm the observations of both Babetto et al., 2020 and Vaquié et al., 2019 who used rat SCs in similar microfluidic culture systems (Vaquié et al, 2019; Babetto et al, 2020). We have shown that the axo-protective observation seen by Babetto et al., 2020 does not rely on myelination status, which was an outstanding question from that study (Babetto et al, 2020). Furthermore, in an advance from previous studies, we have visualised the axo-protective and axon clearance phenomena in the same culture and shown that they are temporally separated, with axon fragmentation and debris clearance by SCs occurring at much later timepoints after axotomy.

Author response: We have now added more in-depth discussion of the similarities and differences between Babetto et al., 2020 and Vaquie et al., 2019 and our approach in the discussion.

Discussion.
Several different culture and experimental conditions preclude direct comparison of our study with both those of Vaquié et al., 2019 and Babetto et al., 2020. These include the use of rat SCs in both prior studies and that Babetto et al., 2020 mixed rat SC with mouse DRG axons; length of time in culture, time points quantified after injury and distance from injury and site of analysis. Babetto et al., 2020 performed axotomy on relatively short term cocultures (6 days in vitro) whereas Vaiquié et al., 2019 cultured for at least 4 weeks and in our case 6 weeks prior to axotomy. Vaiquié et al., 2019 removed nerve growth factor (NGF) prior to laser axotomy and quantified proximally (though also imaged distally) whereas both our study and Babetto et al., 2020 kept NGF in the medium, performed axotomy with a scalpel and quantified more distally and in our case extremely distal, where only individual neurites and no axon bundles were visible. Vaquié et al., 2019 had SCs on both sides of the barrier in the microfluidic chambers whereas we seeded SCs only in the axonal/bottom compartment. Additionally, our cocultures had both forskolin and βNRG1 added to help induce myelination, whereas these factors are not required in rat myelinating cocultures. Finally, it is important to permeabilise myelinated cultures with acetone after fixation, as we did,

© 2023. Published by The Company of Biologists under the terms of the Creative Commons Attribution License (https://creativecommons.org/licenses/by/4.0/).

to visualise the entire axon through heavily myelinated segments otherwise axon integrity cannot be reliably assessed in a quantitative manner (Vaquié et al, 2019; Babetto et al, 2020).

Author response: We would like to add that we showed Claire Jacob, senior author of the Vaquie et al., 2019 study, our manuscript prior to peer review and she offered helpful comments, which we incorporated into the manuscript, which is why she is acknowledged. We have also discussed our findings with Elisabetta Babetto as well.

Overall, the title does not appear to be the most appropriate because the content rather proposes a detailed protocol and gives examples of applications, rather than focusing on the protective versus destructive role of SCs on axons. It also appears to be misleading, as "axo-destructive" appears to suggest a negative role of Schwann cells on axons, whereas SC are rather helpful in clearing degenerative axons, a step which facilitates regeneration.

Author response: We have now changed the title and the focus of the manuscript in line with the reviewer's comments. New title:

A method for mouse myelinating Schwann cell-DRG neuron compartmentalised cocultures: myelination status does not influence the axo-protective effect of Schwann cells after injury.

Author response: We have removed the phrase axo-destructive throughout the manuscript and instead referred to axon fragmentation and axon debris clearance roles of SCs in line with the reviewer's suggestion. Please see track changes manuscript for all instances where this was modified.

The number of biological replicates for each experiment is not always indicated, and if the "n=" represent cultures prepared independently/passaged or wells/cell. It is essential to be rigorous and clearly indicate the number of technical replicates and biological samples throughout the manuscript and provide a thorough description of them. One example is Fig. 4 E were only 10 cells from a single culture appeared to have been imaged. Is this accurate? This aspect is essential to evaluate reproducibility, especially in view of the technical and biological variability.

Author response: We have now added quantification of myelin segments per mm2, percentage of SCs that myelinate and quantification of the interperiodic distance of the myelin formed. This is all included in a new version of TABLE 1. We have discussed this data in the results section as follows.

Quantifying the number of PRX positive myelin segments we found that there were 325.33 ± 12.3 sheaths per mm2, comparable to what has been originally described in rat SC/DRG cocultures and two fold more extensive myelination than recently described compartmentalised rat cocultures models (n=3; Table 1; Eldridge et al, 1987; Vaquié et al, 2019). Furthermore, 25.47 ± 1% of Schwann cells were myelinating in our cultures (n=3; Table 1). To confirm that cocultured myelinated SCs formed compact myelin we performed electron microscopy (EM), which revealed compact myelin formation with multiple myelin wraps and formation of readily visible major dense (MDL) and intraperiod lines (IPL; Fig. 2D). Additionally, we measured the periodicity, i.e., the distance between two adjacent major dense lines, to make sure myelin was compacted. Interperiodic distance was 12.16 ± 0.28 nm, in line with previous reports (n=3; Table 1; Boutary et al, 2021; Fernando et al, 2016; García-Mateo et al, 2018; Giese et al, 1992; Perrot et al, 2007).

Author response: We have discussed the number of cultures used for each quantification in the methods section. See below.

Quantification of Myelination in cocultures

To quantify the number of myelin segments per area, we counted the number of myelin segments for five areas per culture for three cultures and normalised this per mm2. To quantify the percentage of Schwann cells in myelinating cocultures that are actively myelinating, we quantified the number of myelin segments and the number of DAPI-positive nuclei for five areas per culture for three independently prepared cultures. To measure interperiodic distance, we measured at least 10 periods per myelinated fibre for at least three fibres per sample for three separate samples.

© 2023. Published by The Company of Biologists under the terms of the Creative Commons Attribution License (https://creativecommons.org/licenses/by/4.0/).

Quantification of Degeneration
Five images at a distance of between 1.2-1.4mm from the microgroove barrier (the most distal part of the culture that could be imaged) were quantified per culture, taken in comparable locations in each culture. A line was drawn across each image, and each axon crossing this line was either scored as degenerated or intact. Images were blinded prior to quantification. A minimum of three independently prepared cultures were assessed per timepoint for each condition.

Author response: We have removed Fig.4E and instead quoted the data in the results section as follows:

When we quantified this phenomenon, we found that 97.84 ± 1.462% (n=2) of SCs in our cocultures contained mCherry-labelled axonal fragments.

Author response: We apologise as the n number for this experiment was 2 (not 10), with cells in 10 areas quantified throughout all imaging timepoints from each independently prepared culture. We have included the following description in the methods section:

To quantify number of SCs with fragments, each cell was defined as a region of interest and checked for the presence of mCherry positive fragments at all timepoints. Two separate independently prepared cultures and cells in 10 areas per culture were analysed.

Author response: Additionally for Fig. 4B we have now included individual data points from independently prepared cultures.

N numbers are included in all figure legends and always refers to independently prepare cultures/biological replicates.

We have added to the relevant figure legends (Fig.3 and 4 and Table 1) the phrase:
n number refers to independently prepared cultures from separate litters of mice.

Minor comments:

• Does myelination reach axons in the microgrooves (it seems to from 2C, but up to where)? Where is axotomy performed and are axons myelinated where the cut was performed?

Author response: Myelination occasionally reaches the beginning of the microgrooves. We didn't visualise myelination in the DRG cell body compartment. We have added the following detail to the methods section:

Traumatic axotomies were carried out by carefully removing the microfluidic chamber (SND150 and RND150, Xona Microfluidics®) from the Aclar® coverslip using sterile forceps and severing axons with a surgical blade under a light microscope. Axotomies were carried out at the level of the microgroove barrier. To confirm all axons were severed, a second higher cut was performed and axons between the cut sites removed using the surgical blade.

Author response: Given this, we cannot exclude that the odd proximal myelin segment is cut, but the vast majority of axons are not myelinated at the site of cut (lower cut).

• Since the model allows for comparison of aligned vs myelinating SCs, and that both aligned and myelinating SCs seem to slow down degeneration, and that c-JUN is upregulated after in vivo injury, have the authors measured if c-JUN levels increase similarly in both myelinating vs aligned SCs?

Author response: We thank the reviewer for this suggestion. We have now quantified the JUN upregulation after injury in both myelinating and aligned cocultures as well as adding images of JUN upregulation in aligned cocultures. See Fig. 3B-E).

© 2023. Published by The Company of Biologists under the terms of the Creative Commons Attribution License (https://creativecommons.org/licenses/by/4.0/).

Additionally, we noted a strong upregulation of JUN protein in SCs 12 hours after axotomy (Fig. 3B and C). We also saw significant JUN upregulation 12 hours after axotomy in cocultures with aligned SCs (Fig. 3D and E).

Author response: We have decided to focus the manuscript more on the comparison of myelinating versus non-myelinating cocultures, given that we have shown that the axo-protective effect of SCs is independent of myelination status, which is an advance on what is known in the literature. In addition to changing the title, as we have mentioned previously, we have added further characterisation of our aligned cocultures with p75NTR immuno and EM images (Fig.1D and E).

We have
• On clarity:
- In the step-by-step protocol, wording needs to be improved.
Author response: We have substantially edited the step-by-step protocol. Please see track changes document for all specific changes in wording.

- Temperatures for centrifugations are missing.
Author response: We have added temperatures for all centrifugation steps. Please see track changes document

- The MOI described for lentivirus is 2-10 in the protocol but 200 in the legend of Figure 3F.
Author response: The MOI for DRGs was 2-10 and SCs was 200 in Figure 3F. This is described similarly in the extended methods section. DRGs are transduced much more easily than SCs.

We have added the following sentence to the results section to emphasise this point:

Importantly dissociated mouse SCs required a much higher multiplicity of infection (MOI) than dissociated mouse DRGs (see extended methods section).

- Certain citations in the references list are incomplete (i.e. Babetto et al.; Catenaccio et al.,).
Author response: We have updated the reference list.

Reviewer #3 (Significance (Required)):

Significance

The advance for the field proposed by this paper is mostly technical, as it details a new model to be used by the field, of mouse SCs-mouse DRGs in dissociating myelinating cultures. The tested applications allowed the authors to also confirm a protective role for SCs on axonal damage, which was independent from myelination status.

Being a method paper, it is essential that the authors provide clear statements on the number of biological replicates, and technical repeats, as well as a very thorough and accurate description of the methodology.

The model described has similarities with existing models in the field such as Stevens et al., 1998 and Vaquié et al., 2019. To place it in context in a more helpful way, the authors should emphasize on the novelty brought by their protocol compared to existing models. The authors compare their findings to results from Vaquié et al., 2019 and Babetto et al., 2020 that they describe as conflicting, when it seems they rather address different mechanisms of SCs in protection and repair, occurring at different time points.

Audience might be interested in the detailed step by step protocol to use this in vitro model for the applications described, and investigate further why SCs myelination status does not influence their ability to protect from neurodegeneration early on or how to make use of this for neuroprotection studies.

Author response: We have now rephrased the description of Vaquié et al., 2019 and Babetto et al., 2020 studies in line with the reviewer's suggestions. We have now added further discussion of our

© 2023. Published by The Company of Biologists under the terms of the Creative Commons Attribution License (https://creativecommons.org/licenses/by/4.0/).

model in the context of all other models in the field as we have outlined in detail in above responses.

Second decision letter

MS ID#: JOCES/2023/261557
MS TITLE: Schwann cells are axo-protective after injury irrespective of myelination status in mouse Schwann cell/neuron cocultures
AUTHORS: Clara Mutschler, Shaline V Fazal, Nathalie Schumacher, Andrea Loreto, Michael P Coleman, and Peter Arthur-Farraj
ARTICLE TYPE: Tools and Resources

I am happy to tell you that your manuscript has been accepted for publication in Journal of Cell Science, pending standard ethics checks. Where referee reports on this version are available, they are appended below.

© 2023. Published by The Company of Biologists under the terms of the Creative Commons Attribution License (https://creativecommons.org/licenses/by/4.0/).
